# Margin-enriched CD4^+^ central-memory T cells affect macrophages via ANXA1-FPR1 signaling axis to promote tumor progression in intrahepatic cholangiocarcinoma

**DOI:** 10.7150/ijbs.136469

**Published:** 2026-08-11

**Authors:** Zhifeng Jiang, Ao Huang, Runze Miao, Shiyu Zhang, Feiyu Chen, Yang Xu, Jiayan Yan, Lixing Li, Senquan Zhang, Rongkui Luo, Wen Huang, Haixiang Sun, Xinrong Yang, Jian Zhou

**Affiliations:** 1 Department of Liver Surgery and Transplantation, Liver Cancer Institute, Zhongshan Hospital, Fudan University, Key Laboratory of Carcinogenesis and Cancer Invasion (Fudan University), Ministry of Education, 136 Yi Xue Yuan Road, Shanghai 200032, China.; 2 Shanghai Key Laboratory of Organ Transplantation, Zhongshan Hospital, Fudan University, Shanghai 200032, China.; 3 Institute of Biomedical Sciences, Fudan University, Shanghai 200032, China.; 4 State Key Laboratory of Genetic Engineering, Fudan University, Shanghai 200032, China.; 5 Department of Pathology, Zhongshan Hospital, Fudan University, Shanghai 200032, China.

**Keywords:** intrahepatic cholangiocarcinoma (iCCA), immunoenvironment, CD4^+^ central memory T cells, macrophages, ANXA1

## Abstract

Intrahepatic cholangiocarcinoma (iCCA) is the second most prevalent liver cancer with a high mortality and recurrence rate, and remains a poorly understood disease. The tumor margin, as the transition zone between normal tissue and tumor, was not appreciated before. We performed mass cytometry (cytometry by time of flight, CyTOF) on 30 samples from iCCA tumor, paratumor and margin tissue. We found that the number of CD4^+^ central memory T cells (CD4^+^ T_cm_) increased in the margin zone. Single cell RNA-seq (scRNA-seq) further discovered that CD4^+^ T_cm_ cells enriched in the margin zone of iCCA exhibited upregulated Annexin A1 (ANXA1) expression. Fibroblasts recruited CD4^+^ ANXA1^+^ T_cm_ cells via Chemokine (C-C motif) ligand 19 (CCL19)-Chemokine (C-C motif) receptor 7 (CCR7) signaling pathway. The molecular characteristics of CD4^+^ ANXA1^+^ T_cm_ in iCCA were characterized, and the interactions between these T cells and other cells were determined. Patients with enriched CD4^+^ ANXA1^+^ T_cm_ cells in the tumor margin exhibited a worse prognosis. Specifically, CD4^+^ ANXA1^+^ T_cm_ cells could recruit macrophages to the tumor margin and regulate the macrophage polarization via the ANXA1-Formyl Peptide Receptor 1 (FPR1) signaling axis. CD4^+^ ANXA1^+^ T_cm_ cell-activated macrophages could enhance the invasion and proliferation of tumor cells by exerting different cytokines. In conclusion, our study systematically characterized the features and distribution of CD4^+^ ANXA1^+^ T_cm_ cells in the margin zone of iCCA. We investigated the potential mechanisms by which CD4^+^ ANXA1^+^ T_cm_ cells affected tumor progression, and provided novel understanding of the function of these CD4^+^ T_cm_ cells in iCCA.

## Introduction

Intrahepatic cholangiocarcinoma (iCCA), as the second most prevalent primary hepatic tumors, accounts for about 10%-20% of all primary liver cancer, ranking behind hepatocellular carcinoma (HCC)^1^. With increasing patients with hepatitis C or nonalcoholic steatohepatitis, the incidence of iCCA has increased with an average of 4.4% globally over the past decade^2^, ^3^. Patients with iCCA are frequently diagnosed at an advanced stage due to their typically asymptomatic nature in the early stages, which results in poor overall survival rates. Currently, surgical resection remains the most frequent treatment option for iCCA, whereas, 60% patients can experience tumor recurrence or metastasis^4^. Exploring potential mechanisms is important for improving treatment efficiency and preventing iCCA progression.

In previous studies, we identified a tissue-specific immune microenvironment at the margin zone of iCCA. Building upon this, the present study employed an integrated multi-omics approach — determined by the cytometry by time of flight (CyTOF), single-cell RNA sequencing (scRNA-seq), spatial transcriptomics, and bulk RNA-seq—to comprehensively delineate the composition and spatial architecture of immune cell, and underlying mechanisms in the margin zone. Our study found that CD4^+^ T_cm_ cells were enriched at the tumor margin in iCCA, as determined by CyTOF analysis. Significant enrichment of CD4^+^ T_cm_ cells in the tumor margin was correlated with worse prognosis of patients with iCCA. To investigate the potential mechanisms underlying this association, scRNA-seq identified a subset of CD4^+^ T_cm_ cells that exhibit elevated Annexin A1 (ANXA1) expression and remain in a quiescent state in iCCA.

Consistent with these biological properties, these CD4^+^ ANXA1^+^ T_cm_ cells were found to be reprogrammed into a cell-intrinsic dysfunctional state, and recruited by Chemokine (C-C motif) ligand 19+ (CCL19^+^) fibroblasts. These T cells can interact with macrophages through the ANXA1- Formyl Peptide Receptor 1 (FPR1) signaling pathway, thereby playing specific role in iCCA. Macrophages activated by CD4^+^ ANXA1^+^ T_cm_ cells can be considered negative regulators of the immune system, promoting iCCA progression. In summary, CD4^+^ ANXA1^+^ T_cm_ cells may play a role in iCCA progression and could serve as prognostic biomarker and therapeutic target.

## Materials and Methods

Detailed methods of tissue and transcriptomic data processing, data analysis, *in vivo* experiments and *in vitro* experiments can be found in the supplementary methods.

## Results

### The immune landscape in three different regions of iCCA by CyTOF analysis

**Figure 1:**
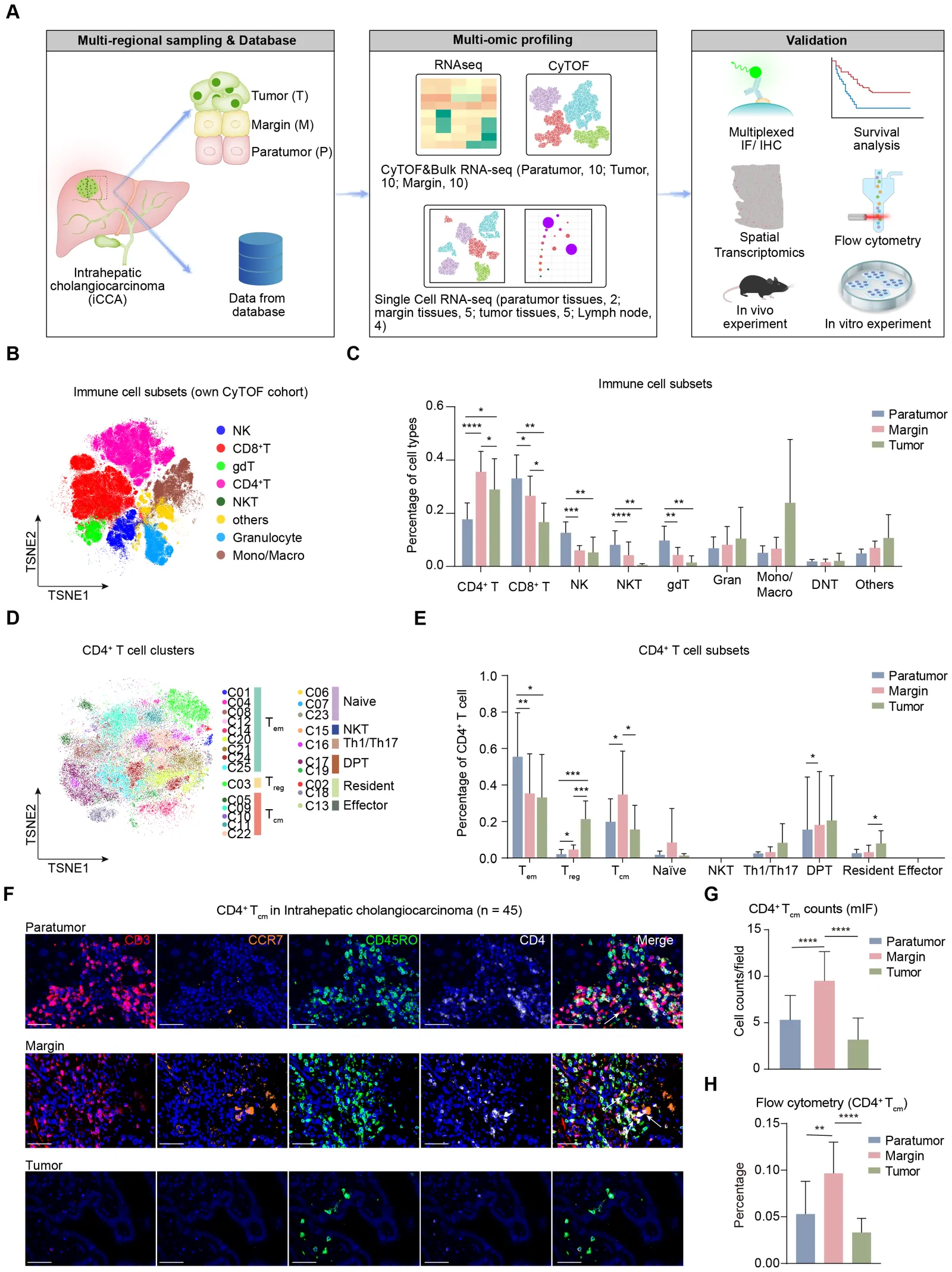
The accumulation of CD4^+^ central-memory T cells (CD4^+^ T_cm_) in the margin zone of intrahepatic cholangiocarcinoma (iCCA). **(A)** A summary of study. The cytometry by time of flight (own CyTOF cohort, 30 samples) and bulk RNA-seq (30 samples) for 10 patients with iCCA were performed. Spatial transcriptomics (public ST database, 2 samples) and scRNA-seq data (CNP0002199, 16 samples) was analyzed. FFPE tissue were subjected to both multiplexed IF staining and IHC staining. The survival analysis was performed to assess the patients' prognosis. Flow cytometry, *in vivo* experiments and *in vitro* experiments were performed to validate the results. **(B and C)** Overview and percentage of immune cell subsets in paratumor, margin and tumor, respectively (CD4^+^ T, Margin vs Paratumor, p < 0.0001, Margin vs Tumor, p < 0.05). **(D and E)** Overview and percentage of CD4^+^ cell subsets in paratumor, margin and tumor, respectively (CD4^+^ T_cm_, Margin vs Paratumor, p < 0.05, Margin vs Tumor, p < 0.05). **(F and G)** mIF staining and accumulation of CD4^+^ T_cm_ in paratumor, margin and tumor, respectively (n = 45; Upper Scale: 500um; Lower Scale: 50um; Margin vs Paratumor, p < 0.0001, Margin vs Tumor, p < 0.0001). **(H)** Flow cytometry of CD4^+^ T_cm_ in paratumor, margin and tumor, respectively (n = 11; Margin vs Paratumor, p <0.01, Margin vs Tumor, p < 0.0001). mIF, multiplexed Immunofluorescence; NK, natural killer cell; Mono/Macro, monocyte or macrophage; NKT, natural killer T cell; Gran, granulocyte; DNT, double negative T cell; T_em_, effector memory T cell; T_reg_, regulatory T cell; T_cm_, central memory T cell; Th1, T helper 1 cell; Th17, T helper 17 cell; DPT, double positive T cell. * p < 0.05, ** p < 0.01, *** p < 0.001, **** p < 0.0001.

To systematically investigate the number of immune cells and the immune signature in human iCCA, we processed fresh tumor, paratumor and tumor margin through CyTOF and bulk RNA-seq. Additionally, we analyzed relative single-cell data from the public database (Figure 1A). For further validation, spatial transcriptomics, bulk RNA-seq analysis of mouse macrophage, IF, IHC and *in vivo* experiment were performed (Figure 1A). We generated CyTOF data from 30 samples (P, 10; M,10; T, 10) obtained from 10 patients diagnosed with iCCA. Detailed clinicopathological and data generation information are provided in Supplementary information (Tables S1 and S2).

All 45 cell clusters from the tumor, paratumor, and marginal zones of 10 patients were shown in TSNE plots (Figure 1B and S1A). To further investigate the immune signature in the tumor margin, we analyzed the proportion of various immune cell types across different tissue samples. The immune cell subsets were further subdivided according to the marker genes of cells (Figure S1B). We analyzed the percentage of cells involved in innate immune response, cells involved in adaptive immune response and other cells, respectively (Figure S1C). Cells involved in adaptive immune response were found to concentrate in the tumor margin (Paratumor vs Margin, p < 0.01; Margin vs Tumor, p < 0.05; Figure S1D). The percentage of cells involved in innate immune response in the tumor margin was less than that in the paratumor (Paratumor vs Margin, p < 0.01; Figure S1D). We counted the myeloid cells, lymphoid cells and others. The percentage of myeloid cells in the tumor margin was less than that in the tumor (Margin vs Tumor, p < 0.05; Figure S1E), The percentage of lymphoid cells in the tumor margin was more than that in the tumor (Margin vs Tumor, p < 0.01; Figure S1E), These data might indicate that there was a specific immune profile in the margin zone of iCCA.

Positional clustering of 8 immune cell subsets was observed in TSNE plot, as follows: natural killer cells (NK), CD8^+^ T cells, γδT cells (gdT), CD4^+^ T cells, natural killer T cells (NKT), Granulocytes, monocytes/macrophages (Mono/Macro), and others (Figure 1B). The immune landscape at the tumor margin was apparently different compared with that in the normal tissue and tumor zone. In particular, the percentage of CD4^+^ T cells at the tumor margin was higher than in the tumor (Margin vs Tumor, p < 0.05) and the paratumor (Paratumor vs Margin, p < 0.0001). This phenomenon was not exhibited by other immune cell subsets (Figure 1C). To better understand how CD4^+^ cells function, we analyzed CD4^+^ T cell subtypes. Across different zones of iCCA, 25 clusters were identified (Figure 1D and S1F).Based on the marker genes of CD4^+^ cells from different clusters (Figure S1G), we classified total CD4^+^ T cells into distinct cell types, including CD4^+^ effector memory T cells (T_em_), CD4^+^ central memory T cells (T_cm_), CD4^+^ naïve T cells (Naive), regulatory T cells (T_reg_), CD4^+^ tissue-resident T cells (Resident), NKT, T helper 1 cells/T helper 17 cells (Th1/Th17), double positive T cells (DPT) and CD4^+^ effector cells (Effector) (Figure 1D). We further analyzed the ratios of each CD4^+^ T cell subsets to total CD4^+^ T cells in the tumor, paratumor, and margin zone, respectively. It was showed that the proportion of CD4^+^ T_cm_ cells in the tumor margin was significantly higher than that in the other two zones (Paratumor vs Margin, p < 0.05; Margin vs Tumor, p < 0.05; Figure 1E).

We next verified the distribution of CD4^+^ T cells and CD4^+^ T_cm_ cells, which were concentrated in the tumor margin areas, defined as a 1000 μm-wide region centered on the margin as described in the previous study^5^. IHC staining exhibited the concentration of CD4^+^ T cells in the margin zone (iCCA, n = 37; Figure S2A-B). The result of multiplexed immunofluorescence (mIF) staining of 45 iCCA margin areas revealed that CD4^+^ T_cm_ were significantly enriched in the tumor marginal areas (p < 0.0001; Figure 1F, 1G, S3A and S3B). The percentage of CD4^+^ T_cm_ cells in the tumor margin was also tested via the flow cytometry (Figure 1H and S2C). In summary, CD4^+^ T_cm_ cells were enriched in the tumor margin area.

### Identification of CD4^+^ T_cm_ in iCCA and the molecular mechanism of CD4^+^ T_cm_ recruitment in the tumor margin area

**Figure 2:**
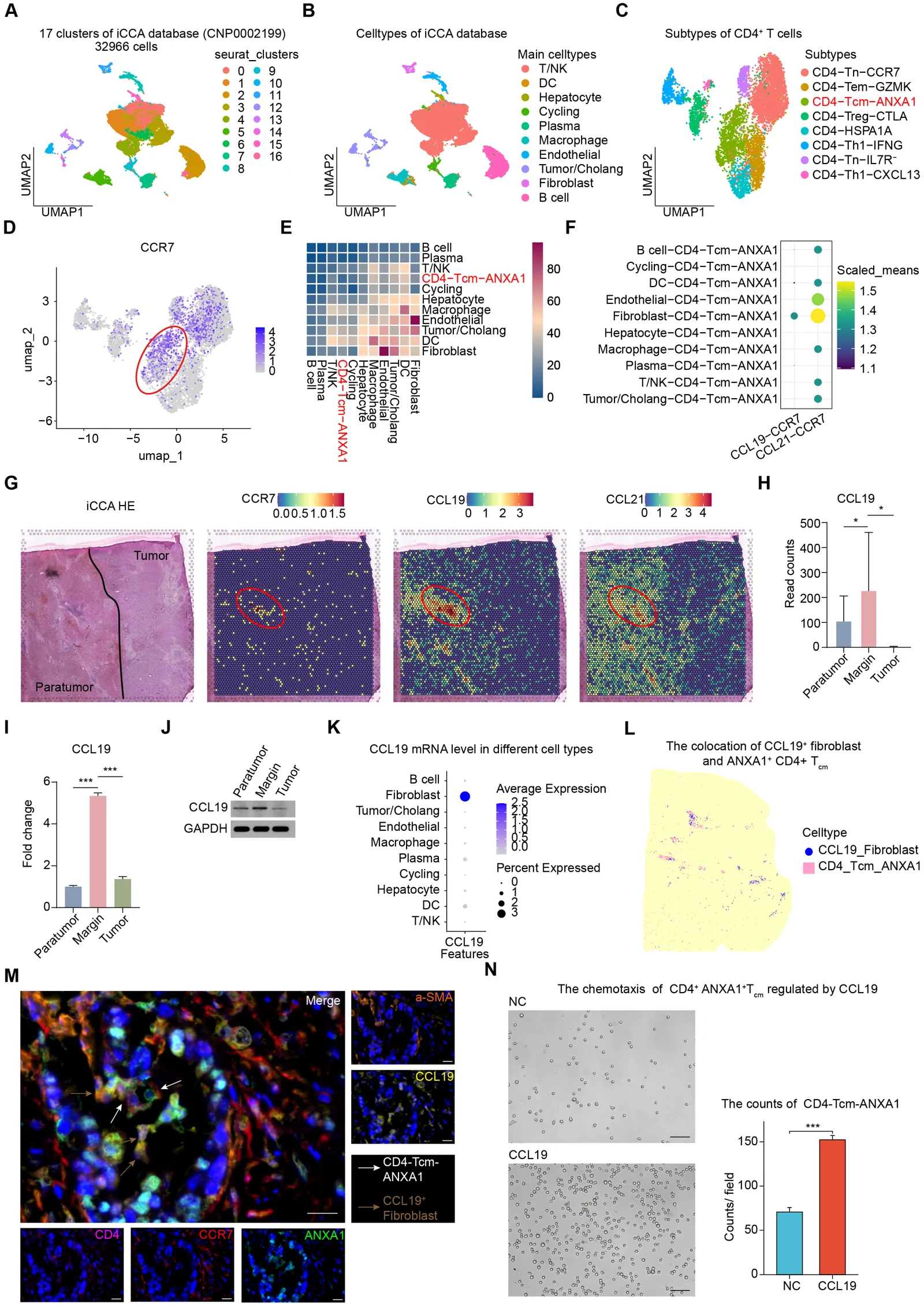
The characterizations of CD4^+^ T_cm_ cells based on public scRNA-seq data (CNP0002199). **(A)** UMAP plot of 17 clusters in the intrahepatic cholangiocarcinoma (iCCA), colored by cluster identity. **(B)** UMAP plot of cell types in the intrahepatic cholangiocarcinoma (iCCA), colored by cluster identity. **(C)** UMAP plot of CD4 T cell types in the intrahepatic cholangiocarcinoma (iCCA), colored by cluster identity. **(D)** UMAP plot of CCR7 expression level in all CD4^+^ T cell subsets **(E)** Potential interactions between CD4^+^ ANXA1^+^ T_cm_ and other cells in margin zone of iCCA via CellphoneDB. **(F)** CCL19/CCL21-CCR7 interaction between CD4^+^ ANXA1^+^ T_cm_ cell and other cells via CellphoneDB. **(G)** CCR7, CCL19, CCL21 mRNA level in the tumor margin based on public spatial transcriptomics. **(H)** Read counts of CCL19 in different tissues (P, M, T) based on bulk RNA-seq (Margin vs Paratumor, p <0.05, Margin vs Tumor, p < 0.05). **(I)** The mRNA level of CCL19 in different tissues (P, M, T) by qRT-PCR analysis (Margin vs Paratumor, p < 0.001; Margin vs Tumor, p < 0.001). **(J)** The protein level of CCL19 in different tissues (P, M, T) by WB analysis. **(K)** The mRNA level of CCL19 in different cell types in margin zone of iCCA based on scRNA-seq. **(L)** The co-location of CD4^+^ ANXA1^+^ T_cm_ and CCL19^+^ fibroblast in margin zone of iCCA based on spatial transcriptomics. **(M)** The co-location of CD4^+^ ANXA1^+^ T_cm_ and CCL19^+^ fibroblast in margin zone of iCCA based on mIF staining (Scale: 20μm). **(N)** Recruitment of CD4+ ANXA1+ Tcm cells through CCR7-CCL19 chemotaxis confirmed by transwell assay. (Scale: 100um; CCL19 vs NC, p < 0.001). T_n_, naive T cell; T_em_, effector memory T cell; T_cm_, central memory T cell; T_reg_, regulatory T cell; Th1, T helper 1 cell; DC, dendritic cell; T/NK, T cell or NK cell; Tumor/Cholang, tumor cell or cholangiocyte; NC, negative control; P, paratumor; M, margin; T, tumor. * p < 0.05, ** p < 0.01, *** p < 0.001, **** p < 0.0001

To further elucidate the role of CD4^+^ T_cm_ cells in iCCA, we analyzed public scRNA-seq database (CNP0002199, totally 32966 cells) containing two paratumor tissues, five tumor tissues, five margin tissues and four lymph nodes. The scRNA-seq data was normalized, and the nFeature, nCount and percent_mt were presented by violin plots (Figure S4A). Using UMAP, 17 clusters were identified and visualized (Figure 2A). The distribution of marker genes associated with different cell types was illustrated in the UMAP plot (Figure S4B). Positional clustering of 10 cell subsets was observed in the UMAP plot, as expected: T/natural killer cells (T/NK), dendritic cells (DC), hepatocyte, cycling cells (Cycling), plasma cells (Plasma), macrophage, tumor cells/cholangiocyte (Tumor/Cholang), fibroblasts, and B cells (Figure 2B). Then, CD4^+^ T cells were subdivided into 11 clusters (Figure S4C). Based on the distribution of marker genes within each cluster, CD4^+^ T cells were classified into 8 subsets (Figure 2C). Based on the study by Zhang et al.^6^, the expression of the selected marker genes for CD4^+^ T_cm_ cells, including CD4, CCR7, TCF7, SELL, LEF1 and ANXA1, was shown in the dotplot (Figure S4D). We found that CD4^+^ T_cm_ cells were enriched in the tumor margin significantly upregulated ANXA1 expression (Figure 2C and S4D), which we later termed CD4^+^ ANXA1^+^ T_cm_ cells.

Chemokine (C-C motif) receptor 7 (CCR7) was the chemokine receptor involved in T cell migration^7^. CCR7 was highly expressed in CD4^+^ ANXA1^+^ T_cm_ cells (Figure 2D). To determine the ligand-receptor interactions between CD4^+^ ANXA1^+^ T_cm_ and other cells, we performed cell-cell interaction analysis (CellPhoneDB). The CellPhoneDB analysis results indicated that CD4^+^ ANXA1^+^ T_cm_ cells might interact with macrophages, endothelial cells, DCs, fibroblasts (Figure 2E). CellPhoneDB analysis revealed that the CCL19-CCR7 and CCL21-CCR7 signaling pathways could play an important role in the recruitment of CD4^+^ ANXA1^+^ T_cm_ cells (Figure 2F). To further explore which pathway played a major role, we analyzed the spatial transcriptomic data following the label transfer workflow of Seurat and then displayed the nCount and nFeature profiles (Figure S5A-B). According to marker genes identified from scRNA-seq data for different CD4^+^ T cell subsets, these subsets were distributed in the margin zone based on Stereo-seq (Figure S5C). ST analysis revealed that these two ligand-receptor pairs are spatially localized, and CCL19 was more expressed than CCL21 at the area where CCR7 was highly expressed (Figure 2G). Furthermore, the bulk RNA-seq data exhibited an upregulated CCL19 expression in the margin zones compared to both the tumor zones (p < 0.05) and the paratumor zones (p < 0.05) (Figure 2H). However, CCL21 expression did not significantly differ among the three zones (Figure S5D). The differential expression of CCL19 was further validated at both the RNA and protein levels using real-time PCR and Western blotting (Figure 2I and J). Based on the scRNA-seq data, we discovered that CCL19 was highly expressed in fibroblasts (Figure 2K). Based on the expression levels of CD4^+^ ANXA1^+^ T_cm_ cells and CCL19^+^ fibroblasts, we defined the top 2.5 correlations % bins and their surrounding bins as cell-high bins (Figure 2L), indicating the co-location of CD4^+^ ANXA1^+^ T_cm_ cells and CCL19^+^ fibroblasts in the margin zones of iCCA. The co-localization of these two cells was verified by mIF staining (Figure 2M). To determine the possibility that fibroblast-derived CCL19 promotes the recruitment of CD4^+^ ANXA1^+^ T_cm_, we performed intratumoral injection of CCL19 or anti-CCR7 antibody in mouse models. According to the previous study^8^, CD4^+^ ANXA1^+^ T_cm_ was labelled by Cd4, Cd62l, Cd44, Ccr7 and Anxa1. We observed more aggregation of CD4^+^ ANXA1^+^ T_cm_ at the invasive margin in the CCL19 treatment group than in the anti-CCR7 antibody group, suggesting that the anti-CCR7 antibody abolished the aggregation of CD4^+^ ANXA1^+^ T_cm_ (Figure S5E). We also found that CCL19^+^ fibroblasts were co-located with HIF1α^+^ cells, suggesting that hypoxia might regulate the recruitment of CCL19^+^ fibroblasts or the secretion of CCL19 in the tumor margin (Figure S6A). Additionally, CCL19 significantly increased chemotaxis of CD4^+^ ANXA1^+^ T_cm_ cells in the transwell assay, as indicated by the higher cell counts than in the control (Figure 2N). In sum, CCL19^+^ fibroblasts secreted CCL19 to recruit CD4^+^ T_cm_ cells to the tumor margin.

### Hypoxia upregulating the concentration of CD4^+^ ANXA1^+^ T_cm_ in tumor margin via HIF1α-FOSL2-ANXA1 axis

**Figure 3:**
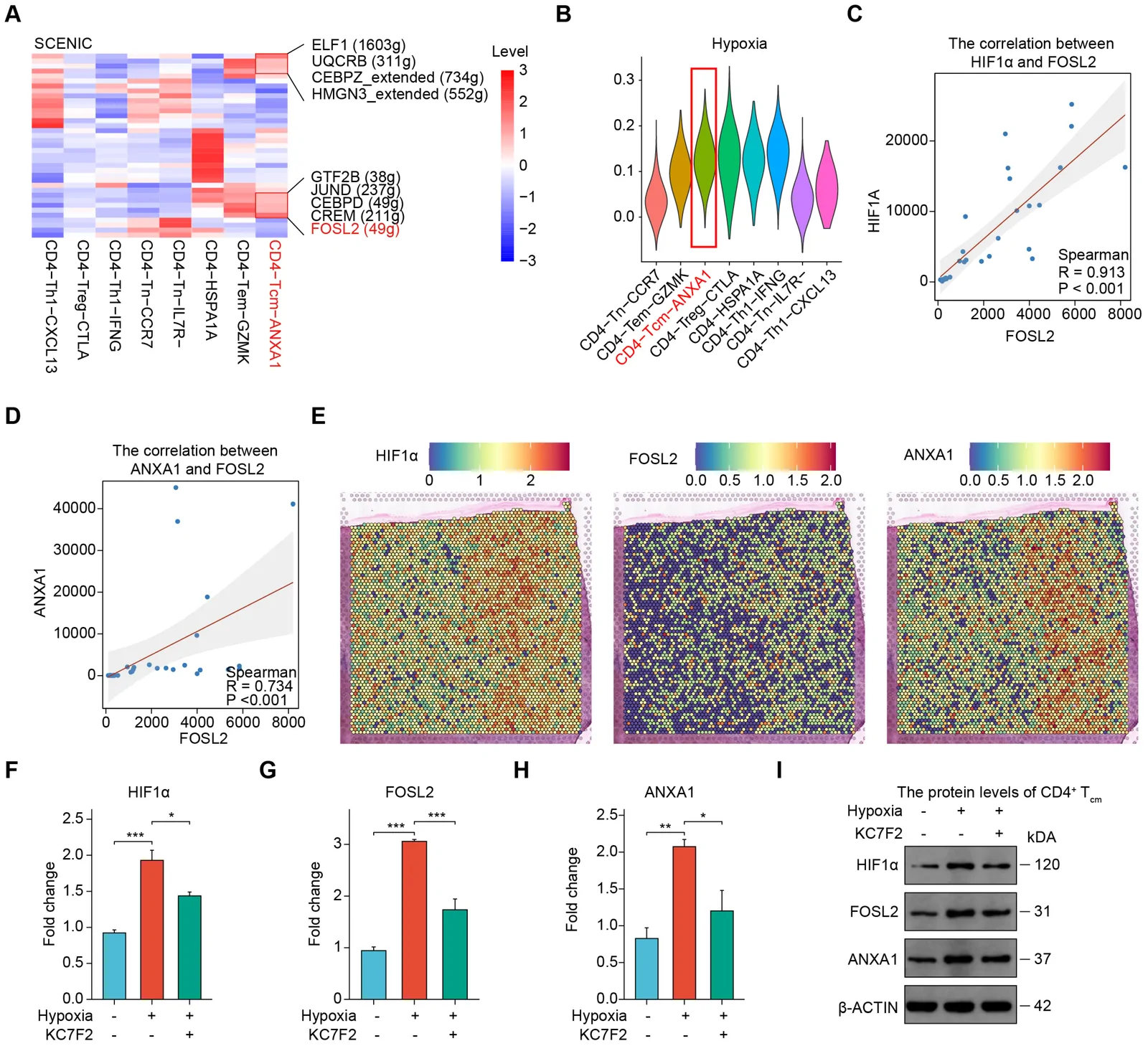
CD4^+^ ANXA1^+^ T_cm_ cells governed by HIF1α-FOSL2 signaling pathway in margin zone of iCCA. **(A)** Critical nodes of CD4^+^ T cell subsets predicted SCENIC analysis. **(B)** ssGSEA analysis of CD4^+^ ANXA1^+^ T_cm_ cell in the margin zone of iCCA. **(C)** The correlation analysis between HIF1α and FOSL2 (Spearman, R = 0.913, p < 0.001). **(D)** The correlation analysis between FOSL2 and ANXA1 (Spearman, R = 0.734, p < 0.001). **(E)** HIF1α, FOSL2, ANXA1 mRNA level in the tumor margin based on spatial transcriptomics. **(F)** qRT-PCR analysis of the expression of HIF1α in CD4^+^ T_cm_ cells (Margin vs Paratumor, p <0.001, Margin vs Tumor, p < 0.05). **(G)** qRT-PCR analysis of the expression of FOSL2 in CD4^+^ T_cm_ cells (Margin vs Paratumor, p <0.001, Margin vs Tumor, p < 0.001). **(H)** qRT-PCR analysis of the expression of ANXA1 in CD4^+^ T_cm_ cells (Margin vs Paratumor, p <0.01, Margin vs Tumor, p < 0.05). **(I)** WB analysis of the expression of proteins involved in HIF1α-FOSL2-ANXA1 signaling pathway in CD4^+^ T_cm_ cells. T_n_, naive T cell; T_em_, effector memory T cell; T_cm_, central memory T cell; T_reg_, regulatory T cell; Th1, T helper 1 cell. Figure 3F-H: blue bar, NC group; red bar, hypoxia-treated group; green bar, hypoxia and HIFα-inhibitor group. * p < 0.05, ** p < 0.01, *** p < 0.001, **** p < 0.0001

We used SCENIC to identify transcription factors (TFs) that could regulate CD4^+^ T cell heterogeneity. It was found that specific TFs, including FOSL2, JUND, CREM, CEBPD, GTF2B, ELF1, UQCRB, CEBPZ_extended, and HMGN3_extended, were more active in CD4^+^ ANXA1^+^ T_cm_ cells compared with other CD4^+^ T cells based on the scRNA-seq data (Figure 3A). Among these TFs, FOSL2 was the most significantly upregulated (Figure 3A). In a previous study, we detected that the hypoxia-related pathway was significantly enriched in the margin zones^9^. It was reported that the tissue microenvironment could shape CD4^+^ memory T cell phenotypes^10^. Accordingly, we hypothesized that a hypoxic environment was associated with CD4^+^ ANXA1^+^ T_cm_ cells. The hypoxic score of CD4^+^ T cell subsets was performed via ssGSEA, and we found CD4^+^ ANXA1^+^ T_cm_ cells have the third-highest hypoxic score among all CD4^+^ T cell subpopulations (Figure 3B). The mRNA level of ANXA1 in the margin zones was higher than that in tumor and paratumor zones (Figure S7A). The correlations between HIF1α and FOSL2 and between FOSL2 and ANXA1 were predicted based on the bulk RNA-seq data (Figure 3C- D). The co-localization of HIF1α, FOSL2 and ANXA1 at the tumor margin was demonstrated by ST analysis (Figure 3E). Additionally, HIF1α, FOSL2 and ANXA1 expression were detected at both the RNA and protein levels via qRT-PCR and WB, respectively (Figure 3F-H). Compared to the non-treated CD4^+^ T_cm_ cells, RNA expressions of HIF1α, FOSL2, and ANXA1 were significantly upregulated (p < 0.01) in hypoxia-treated CD4^+^ T_cm_ cells. Similar results were observed at the protein level (Figure 3I). The HIF1α-FOSL2 signaling pathway promoted ANXA1 expression, which was blocked by KC7F2. The HIF1α-FOSL2-ANXA1 signaling pathway was verified in mouse CD4^+^ T cells (Figure S7B-E). In conclusion, the hypoxic microenvironment activated the HIF1α-FOSL2-ANXA1 pathway in CD4^+^ T_cm_ cells, and induced ANXA1 expression.

### The characteristic and biofunction of CD4^+^ ANXA1^+^ T_cm_ in the tumor margin

**Figure 4:**
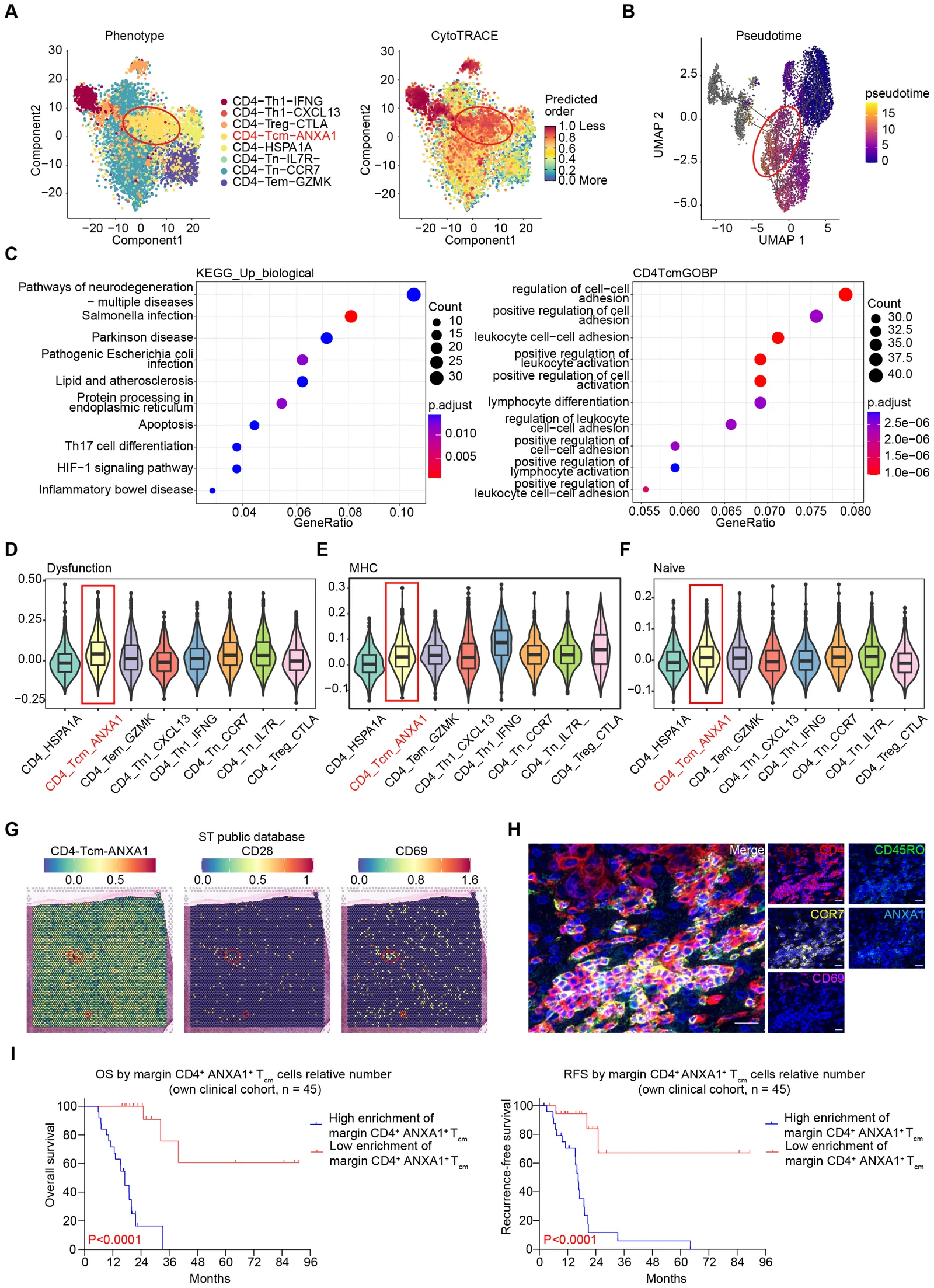
The state of CD4^+^ ANXA1^+^ T_cm_ cells and the biofunction of CD4^+^ ANXA1^+^ T_cm_ cell enrichment based on scRNA-seq and spatial transcriptomics data. **(A)** CytoTRACE analysis of all CD4^+^ T cell subsets in paratumor, margin and tumor based on public scRNA-seq. **(B)** Pseudotime ordering of CD4^+^ ANXA1^+^ T_cm_ cells in all CD4^+^ T cell subsets (All cells were colored by pseudotime score. Color purple indicates the beginning, whereas color yellow indicates the end.). **(C)** KEGG and GO analysis of CD4^+^ ANXA1^+^ T_cm_ cells. **(D)** Dysfunction score of CD4^+^ ANXA1^+^ T_cm_ cells via ssGSEA analysis. **(E)** MHC_features score of CD4^+^ ANXA1^+^ T_cm_ cells via ssGSEA analysis. **(F)** Naive score of CD4^+^ ANXA1^+^ T_cm_ cells via ssGSEA analysis. **(G)** The level of CD4^+^ ANXA1^+^ T_cm_ cells, CD28 and CD69 based on spatial transcriptomics data. **(H)** mIF staining of activated CD4^+^ ANXA1^+^ T_cm_ cells in the margin zones. **(I)** Overall survival (OS) and Recurrence-free survival (RFS) of different enrichment of CD4^+^ ANXA1^+^ T_cm_ cells in the tumor margin in iCCA (n = 45; OS, p < 0.0001; RFS, p < 0.0001). T_n_, naive T cell; T_em_, effector memory T cell; T_cm_, central memory T cell; T_reg_, regulatory T cell; Th1, T helper 1 cell; KEGG, Kyoto Encyclopedia of Genes and Genomes; GO, Gene Ontology; BP, Biological Process. * p < 0.05, ** p < 0.01, *** p < 0.001, **** p < 0.0001.

The differentiation potential of T cell subsets was analyzed using CytoTRACE, and CD4^+^ ANXA1^+^ T_cm_ cells exhibited a certain degree of differentiation capacity (Figure 4A). Using pseudotime analysis of scRNA-Seq data, we traced the lineage specification of CD4^+^ T cell subsets and found that CD4^+^ ANXA1^+^ T_cm_ cells represented an intermediate state in CD4^+^ T cell development (Figure 4B). In general, memory T cells played a key role in inhibiting tumor growth and progression^11^, ^12^. The gene features and biofunctions of CD4^+^ T_cm_ cells in the marginal zones were further explored using scRNA-seq data. The DEGs in CD4^+^ ANXA1^+^ T_cm_ cells were enriched in apoptosis-, immunity- and hypoxia-related signaling pathway via KEGG and GO analysis (Figure 4C). According to previously reported single-cell gene sets of T cell function, it was found that CD4^+^ ANXA1^+^ T_cm_ cells were most likely in a dysfunctional, naïve, and non-activated state (Figure 4D-F), which could, in part, explain why CD4^+^ ANXA1^+^ T_cm_ cells did not inhibit tumor progression. In iCCA, RNA gene expression was highly tissue-specific, as indicated by its expression across the three zones (Figure S8A-B). Using bulk RNA-Seq data, CIBERSORT was used to estimate the relative proportions of immune cell subtypes. The proportion of CD4^+^ T cells in the margin zones was higher than that in the tumor zone (p < 0.01; Figure S8C), and percentage of CD4^+^ T cells in paratumor zones was also higher than that in tumor zone (p < 0.05; Figure S8C), consist with CyTOF results. The proportion of CD4^+^ memory T cells (resting) in the margin zones was higher than that in the tumor zone (p < 0.0001; Figure S8D), suggesting that CD4^+^ T_cm_ cells in the margin zones could be resting. To further validate these findings, we analyzed spatial transcriptomics data and performed mIF staining (Figure 4G-H). It was reported that CD28 promoted the activation and proliferation of T cell^13^. CD69 was well-established as an early activation antigen of immune cells^14^. We found that the spots enriched with CD4^+^ ANXA1^+^ T_cm_ cells in margin zones rarely expressed genes (CD28 and CD69) based on 10X ST data, suggesting that CD4^+^ ANXA1^+^ T_cm_ in the margin zones was in resting state (Figure 4G). Furthermore, we also found that mRNA expression of CD69 and CD28 was not high in bins enriched for CD4^+^ ANXA1^+^ T_cm_ cells, as indicated by the data of Stereo-seq (Figure 2L, S4C and S8E). Similar results were obtained through mIF staining (Figure 4H). Activated CD4^+^ ANXA1^+^ T_cm_ (CD69+ CD4+ ANXA1+) cells were infrequently detected in the marginal zones of iCCA (Figure 4H). The proportion of activated CD4^+^ T_cm_ in all CD4^+^ T_cm_ in margin zones was significantly lower than that in the tumor zones, and was the same as that in paratumor zones according to the results of flow cytometry (Tumor vs Margin, p < 0.001; Tumor vs Paratumor, p < 0.001; Figure S9A). By Oxygen Consumption Rate (OCR) and Extracellular Acidification Rate (ECAR) measured, we found that CD4^+^ ANXA1^+^ T_cm_ were classified as quiescent (Figure S9B-C). Similar result was obtained by ELISA assay (Figure S9D).

Multiple existing studies have demonstrated that CD4^+^ T_cm_ cells might be associated with tumor prognosis, however, a similar phenomenon has not been reported in iCCA. Survival analysis revealed that patients with more CD4^+^ T cells in the margin zone had a worse prognosis compared with patients with fewer CD4^+^ T cells (p < 0.05; Figure S10A). Furthermore, we performed survival analyses of Overall Survival (OS) and Recurrence-Free Survival (RFS) in patients with iCCA (Figure 4I, S11A-B). Patients with high CD4^+^ T_cm_ cell enrichment in the margin exhibited a worse prognosis than those with low CD4^+^ T_cm_ cell enrichment (OS, p < 0.0001; RFS, p < 0.0001).

In conclusion, CD4^+^ ANXA1^+^ T_cm_ cells enriched in the margin zones of iCCA were in a resting state and associated with poor clinical outcomes in patients. In clinical settings, enrichment of CD4^+^ ANXA1^+^ T_cm_ at the tumor margin was emerging as a potential biomarker for iCCA, a decrease in this enrichment markedly improved prognosis.

### Recruitment and polarization of macrophages regulated by CD4^+^ ANXA1^+^ T_cm_ via ANXA1-FPR1 signaling axis

**Figure 5:**
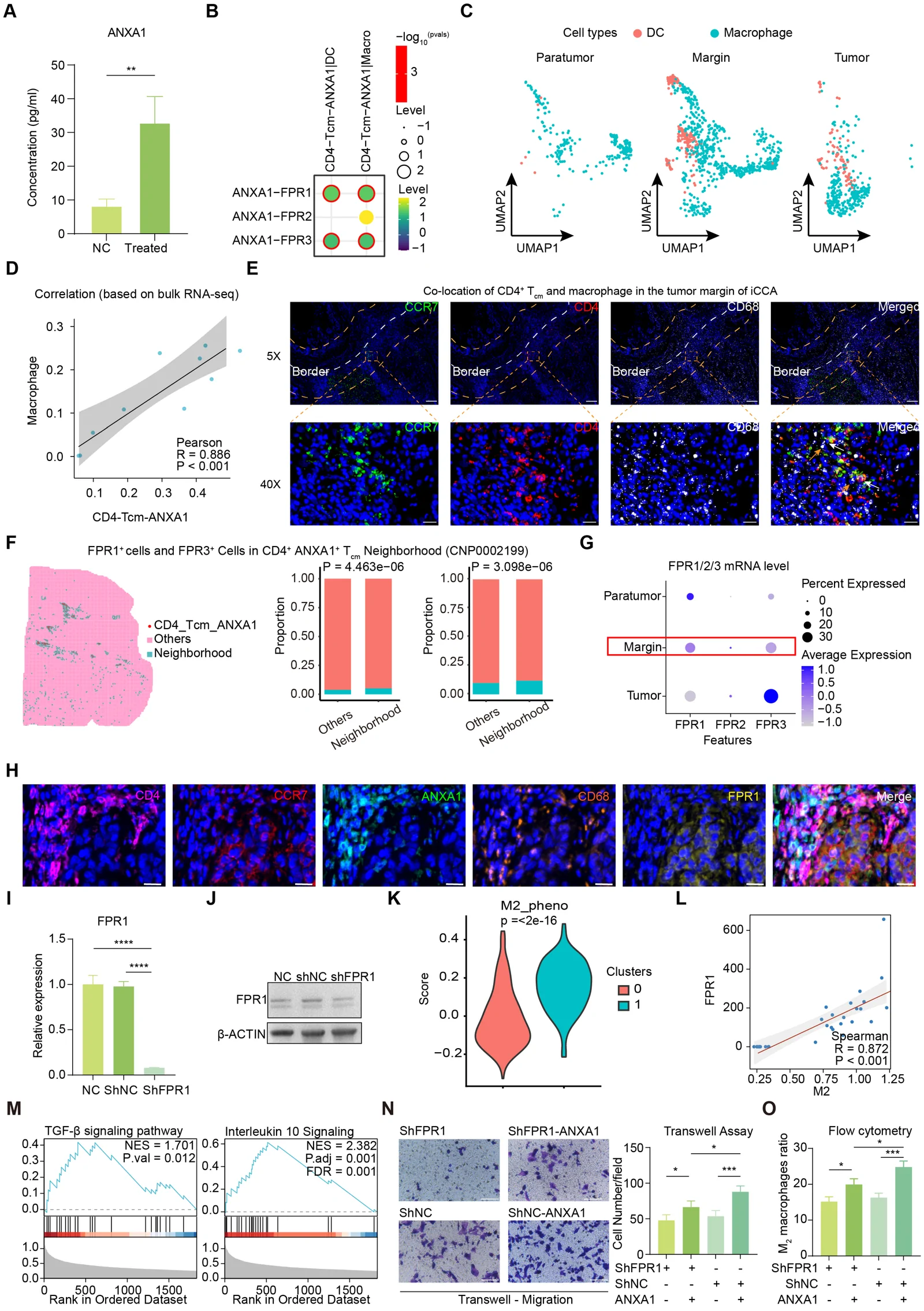
The recruitment and polarization of macrophages regulated by CD4^+^ ANXA1^+^ T_cm_ cells via ANXA1-FPR1 signaling pathway. **(A)** Expression of ANXA1 released by CD4^+^ ANXA1^+^ T_cm_ cells determined by ELISA (NC: the adjacent non-tumor liver tissue group, Treated: the tumor margin group). **(B)** Potential interactions between CD4^+^ ANXA1^+^ T_cm_ cells and other cells via ANXA1-FPR signaling pathway. **(C)** UMAP plot of dendritic cells and macrophages in different zones of iCCA. **(D)** Correlation of macrophages and CD4^+^ ANXA1^+^ T_cm_ cells in the margin zone in iCCA (Pearson, R = 0.886, p < 0.001). **(E)** Co-location of CD4^+^ ANXA1^+^ T_cm_ cells and macrophages validated by immunofluorescence staining (Upper Scale: 200μm; Lower Scale: 20μm). **(F)** The distribution of FPR1^+^ macrophages and FPR3^+^ macrophages in CD4^+^ ANXA1^+^ T_cm_ neighborhood and other tissues (FPR1^+^ cells, Neighborhood vs Other, p = 4.463e-06; FPR3^+^ cells, Neighborhood vs Other, p = 3.098e-06). **(G)** mRNA levels of FPR1, FPR2 and FPR3 in different tissues of iCCA based on scRNA-seq. **(H)** Co-location of CD4^+^ ANXA1^+^ T_cm_ cells and FPR1^+^ macrophages validated by immunofluorescence staining (Scale: 20μm). **(I)** mRNA FPR1 expression in Control, shNC and shFPR1 groups, respectively (ShFPR1 vs ShNC, p < 0.0001; ShFPR1 vs NC, p < 0.0001). **(J)** Protein FPR1 expression in Control, shNC and shFPR1 groups, respectively. **(K)** The M2-type scores of Cluster 0 and Cluster 1 in margin zones of iCCA based on scRNA-seq (Cluster 1 vs Cluster 0, p =< 2e-16). **(L)** The correlation between FPR1 and M2-type macrophage (Spearman, R = 0.872, p < 0.001). **(M)** The GSEA analysis of M2-type macrophage (Cluster 1). **(N)** Migration ability of macrophages and ANXA1-stimulated macrophages assessed by the transwell assay (Scale: 100μm; ShNC-ANXA1 vs ShFPR1-ANXA1, p < 0.05; ShNC-ANXA1 vs ShNC, p < 0.001; ShFPR1-ANXA1 vs ShFPR1, p < 0.05). **(O)** Phenotypic analysis of macrophages stimulated by ANXA1 via flow cytometry (ShNC-ANXA1 vs ShFPR1-ANXA1, p < 0.05; ShNC-ANXA1 vs ShNC, p < 0.001; ShFPR1-ANXA1 vs ShFPR1, p < 0.05). As neighboring bins are not spatially independent, P-values in (F) reflect relative enrichment and should not be read as exact significance levels. T_cm_, central memory T cell; NC, negative control; DC, dendritic cell. * p < 0.05, ** p < 0.01, *** p < 0.001, **** p < 0.0001.

As mentioned above, ANXA1 was identified as the specific gene in CD4^+^ T_cm_ cells in the margin zones. To identify the mechanism by which ANXA1 regulated the progression of iCCA, we first determined whether ANXA1 was released from CD4^+^ ANXA1^+^ T_cm_ cells into the extracellular space. CD4^+^ T_cm_ cells isolated from the tumor margin secreted more ANXA1 protein than CD4^+^ T_cm_ cells isolated from adjacent non-tumor liver tissues from the same iCCA patients through ELISA (p < 0.01, Figure 5A). We further investigated all potential intercellular communication pathways by analyzing ligand-receptor pair expression using CellPhoneDB. We found that CD4^+^ ANXA1^+^ T_cm_ cells were most likely to interact with DC and macrophage via ANXA1-FPR1/2/3 signaling pathway (Figure 5B). The proportion of macrophages in the tumor margin was higher than the proportion of DC in the tumor margin (Figure 5C and S12A). We used the ssGSEA score to quantify the activity or enrichment levels of immune cells in the samples. The enrichment of CD4^+^ ANXA1^+^ T_cm_ cells correlated with that of macrophages based on the bulk RNA-seq data (Figure 5D). Additionally, mIF staining demonstrated that CD4^+^ ANXA1^+^ T_cm_ cells were co-localized with macrophage in the tumor margin (Figure 5E). We found that the percentage of macrophage in CD4^+^ ANXA1^+^ T_cm_ neighborhood was more than that in others (Figure S12B). The results suggested the involvement of local cell-cell interaction between macrophage and CD4^+^ ANXA1^+^ T_cm_. The proportions of FPR1^+^ macrophages in the CD4^+^ ANXA1^+^ T_cm_ neighborhood were significantly higher than those in other neighborhood via the analysis of Stereo-seq (Figure 5F). Furthermore, the proportions of FPR3^+^ macrophages in the CD4^+^ ANXA1^+^ T_cm_ neighborhood were significantly higher than those in other neighborhoods (Figure 5F). The FPR1^+^ and FPR3^+^ macrophage-enriched bins distributed around the CD4^+^ ANXA1^+^ T_cm_-enriched bins (Figure S12C-E). To determine the major receptor of ANXA1, FPR1, FPR2, and FPR3 expression was displayed in a dot plot based on scRNA-seq data (Figure 5G). The FPR1 expression was highest in the margin zones than other two zones (Figure 5G). We further demonstrated that CD4^+^ ANXA1^+^ T_cm_ cells were co-localized with FPR1^+^ macrophage in the tumor margin by mIF (Figure 5H). To further elucidate the role of the ANXA1-FPR1 signaling pathway in the interaction between macrophage and CD4^+^ ANXA1^+^ T_cm_ cells, FPR1-knockdown THP-1 cell lines were constructed. The RNA and protein levels of FPR1 were detected by qRT-PCR and WB (Figure 5I-J).

We found CD163 highly expressed on macrophages at the tumor margin (Figure S12F). Additionally, the immunosuppressive and chemokine-related scores of macrophages in margin zones were significantly higher than those in paratumor zones (Figure S12G-H). To figure out the effect of the interactions between ANXA1 and FPR1 on macrophage, we further subdivided the macrophages into two clusters (cluster 0 and 1; Figure S12I). The levels of FPR1 and M2-type macrophage-related marker genes (such as CD163) in Cluster 1 were higher than those in Cluster 0, suggesting that Cluster 1 was an M_2_-type macrophage with a high level of FPR1 expression (Figure S12J). The M2 macrophage phenotype-related scores of Cluster 1 were higher than that of Cluster 0 (Figure 5K). The FPR1 was found to be highly correlated with M_2_-type macrophages (Figure 5L). The genes of Cluster 1 were enriched in immunosuppressive pathways, including TGF-β signaling pathway and IL-10 signaling pathway (Figure 5M). ANXA1 has been reported to stimulate macrophage chemotaxis and is associated with macrophage polarization 15. Subsequently, we verified the effect of the ANXA1-FPR1 signaling pathway on macrophage migration using a Transwell experiment (Figure 5N). ANXA1 significantly promoted the migration abilities of macrophages (p < 0.001), and the inhibition of FPR1 attenuated the promotion effect of ANXA1 on macrophage migration (p < 0.05). Flow cytometry analysis demonstrated that ANXA1 significantly promoted the M2-type polarization of macrophages (p < 0.001), and the inhibition of FPR1 attenuated the promotion effect of ANXA1 on macrophage polarization (p < 0.05; Figure 5O and S13A). To elucidate the function of ANXA1, we constructed an siANXA1 CD4^+^ T_cm_ cell line and validated the knockdown via Western blot (Figure S14A). Through co-culture experiments and Transwell chamber assays, we demonstrated that ANXA1 regulates the chemotaxis and polarization of macrophages (Figure S14B-C). ARG1 and CD206 expression in Ac2-26-stimulated macrophages was significantly upregulated compared to that in normal macrophages, which was suppressed by HCH6-1 (p < 0.001; Figure S15A). In a previous study, TGF-β and IL-10 were considered immunosuppressive cytokines^16^, ^17^.

Interleukin 6 (IL-6) increased in immunosuppression mediated by macrophages and dendritic cells^18^, ^19^. VEGF could promote tumor growth and angiogenesis^20^. ANXA1-stimulated macrophages secreted more immunosuppressive chemokines (including IL-10 and TGF-β), IL-6 and VEGF than non-treated macrophages (IL-10, p < 0.01; TGF-β, p < 0.05; IL-6, p < 0.01; VEGF, p < 0.01). Furthermore, the inhibition of FPR1 could reduce the promotion effect of ANXA1 on the secretory capacity of macrophages (p < 0.05; Figure S15B-D). These cytokines were primarily secreted by M_2_-type macrophages, and could promote tumor development and create an immunosuppressive microenvironment. Similar results were obtained for the wound-healing assay and mIF staining in RAW264.7 (Figure S16A - B). The bulk RNA-seq was performed on non-treated RAW264.7 and Anxa1-stimulated RAW264.7 to explore the mechanism of macrophage migration and polarization (Figure S16C). In general, Anxa1-upregulated genes were enriched in macrophage differentiation, migration and chemotaxis through the GO analysis. Additionally, these genes were enriched in M2 macrophage polarization-related signaling pathways (including the JAK-STAT signaling pathway^21^, Toll-like signaling pathway^22^, and TGF-β signaling pathway^23^) via the KEGG analysis (Figure S16D). To further verify the role of the ANXA1-FPR1 signaling pathway in the recruitment and polarization of macrophages, we isolated and cultured the murine bone marrow derived macrophages (BMDM), and performed *in vitro* experiments employing murine BMDM. We successfully identified F4/80 and DAPI on BMDM through mIF (Figure S17A). Anxa1-stimulated BMDM secreted more immunosuppressive chemokines (including Il-10 and Tgf-β) and Vegf than non-treated BMDM (Il-10, p < 0.001; Tgf-β, p < 0.001; Vegf, p < 0.001). The inhibition of Fpr1 could reduce the promotion effect of Anxa1 on the secretory capacity of macrophages (Il-10, p < 0.01; Tgf-β, p < 0.05; Vegf, p < 0.001; Figure S17B). Flow cytometry analysis demonstrated that ANXA1 significantly promoted the M2-type polarization of BMDM (p < 0.001), and the inhibition of FPR1 attenuated the promotion effect of ANXA1 on BMDM polarization (p < 0.001; Figure S17C). Arg1 and Cd163 expression in Ac2-26-stimulated BMDM was obviously more than that in BMDM, which was suppressed by Fpr1-inhibitor (Figure S17D). The chemotactic effect of ANXA1 was determined via *in vivo* experiment and mIF staining (Figure S17E).

In summary, CD4^+^ ANXA1^+^ T_cm_ cells at the tumor margin promoted macrophage recruitment and polarization via the ANXA1-FPR1 signaling pathway.

### ANXA1-stimulated macrophages promoting the progression of iCCA

**Figure 6:**
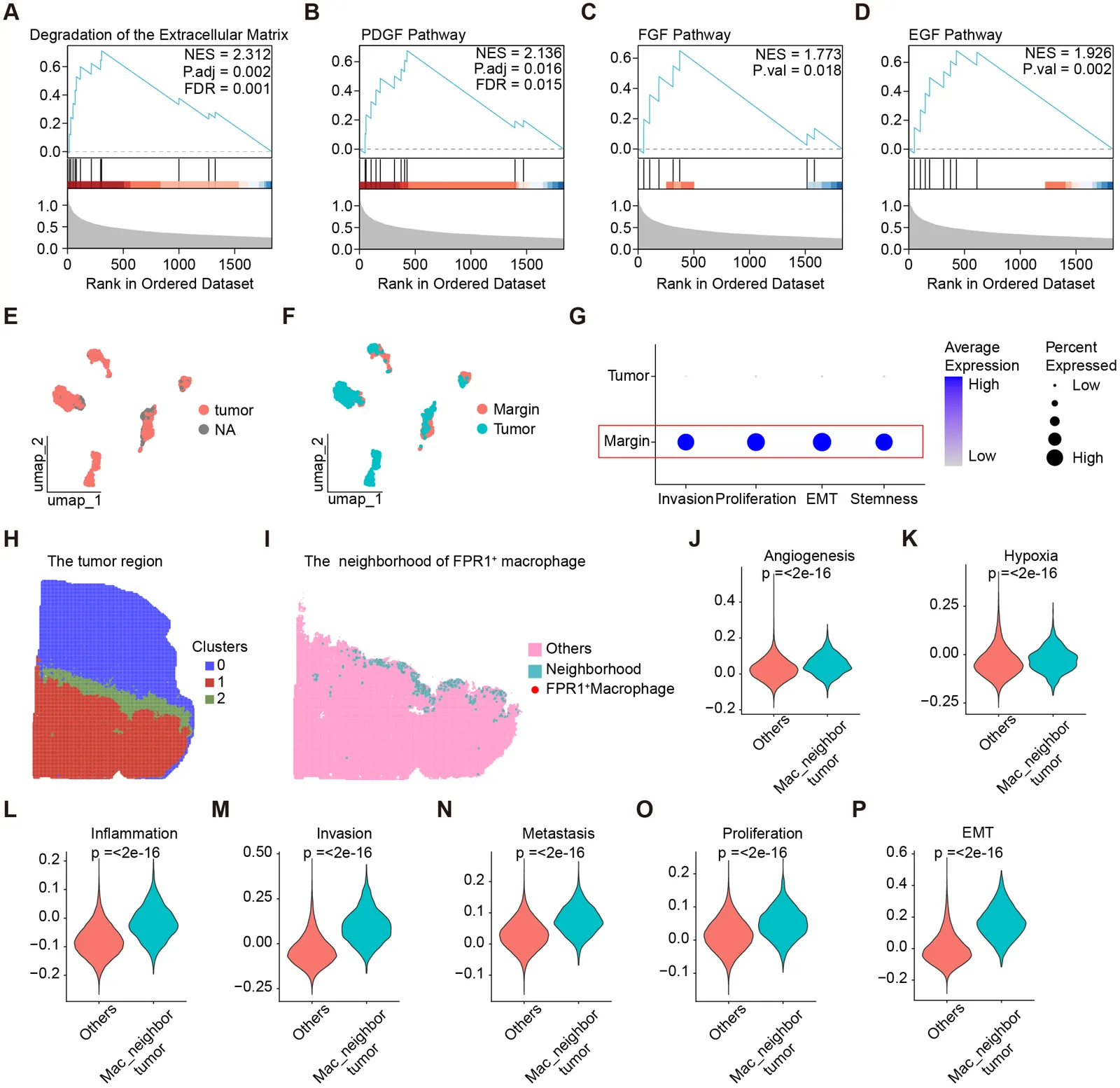
Spatial characteristics of tumor cells in different regions of iCCA **(A to D)** The GSEA analysis of M2-type macrophage (Cluster 1). **(E)** The UMAP plot of tumor cells and non-tumor cells. **(F)** The UMAP plot of tumor cells in different zones of iCCA. **(G)** The tumor-related scores of tumor cells in different zones of iCCA. **(H)** The regions of margin zones in iCCA based on ST. **(I)** The FPR1^+^ macrophages neighborhoods and other neighborhoods based on ST. **(J)** The Angiogenesis scores of tumor cells in FPR1^+^ macrophages and other neighborhoods, respectively (Mac_neighbor tumor vs Others, p =< 2e-16). **(K)** The Hypoxia scores of tumor cells in FPR1^+^ macrophages and other neighborhoods, respectively (Mac_neighbor tumor vs Others, p =< 2e-16). **(L)** The Inflammation scores of tumor cells in FPR1^+^ macrophages and other neighborhoods, respectively (Mac_neighbor tumor vs Others, p =< 2e-16). **(M)** The Invasion scores of tumor cells in FPR1^+^ macrophages and other neighborhoods, respectively (Mac_neighbor tumor vs Others, p =< 2e-16). **(N)** The Metastasis scores of tumor cells in FPR1^+^ macrophages and other neighborhoods, respectively (Mac_neighbor tumor vs Others, p =< 2e-16). **(O)** The Proliferation scores of tumor cells in FPR1^+^ macrophages and other neighborhoods, respectively (Mac_neighbor tumor vs Others, p =< 2e-16). **(P)** The EMT scores of tumor cells in FPR1^+^ macrophages and other neighborhoods, respectively (Mac_neighbor tumor vs Others, p =< 2e-16).

To further investigate the role of CD4^+^ ANXA1^+^ T_cm_ cells in tumor progression, we performed the biofunction of M_2_-type macrophages which were likely to interact with CD4^+^ ANXA1^+^ T_cm_ cells in the margin zones via GSEA analysis (Figure 6A-D). M_2_-type macrophages were associated with the tumor cells' invasion (TGF-β signaling pathways; Figure 5M) and tumor proliferation (PDGF, FGF and EGF signaling pathways; Figure 6B-D). To identify putative malignant tumor cells present in iCCA samples, we subjected the Tumor/Cholang cells to InferCNV analysis (Figure 6E). The tumor cells in the margin and tumor zones were shown in the UMAP plot (Figure 6F). According to the tumor-related gene sets (http://biocc.hrbmu.edu.cn/CancerSEA), we observed that the invasion-, proliferation-, stemness- and EMT-related scores of tumor cells in the margin zones were higher than those in the tumor zones (Figure 6G). Based on these results, we found that macrophages interacting with CD4^+^ ANXA1^+^ T_cm_ cells could significantly promote the invasion, proliferation and migration of tumor cells in the margin zones of iCCA. Three clusters (Cluster 0, 1 and 2) were identified by BANKSY analysis, suggesting that tumor cells in the tumor margin region differed from those in the tumor region (Figure 6H). Based on these above results and Stereo-seq analysis, FPR1^+^ macrophages were enriched in the tumor margin region (Figure 5F and 6I). Furthermore, we compared the biofunctions between tumor cells in the FPR1^+^ macrophage neighborhood and those in others (Figure 6J-P). All of the angiogenesis-, hypoxia-, inflammation-, invasion-, metastasis-, proliferation- and EMT-related scores of tumor cells in the FPR1^+^ macrophage neighborhood were higher than those in others (Figure 6J-P). Combined with scRNA-seq and Stereo-seq results, FPR1^+^ macrophages could promote the malignant progression and metastasis of tumor cells in the tumor margin zone.

**Figure 7:**
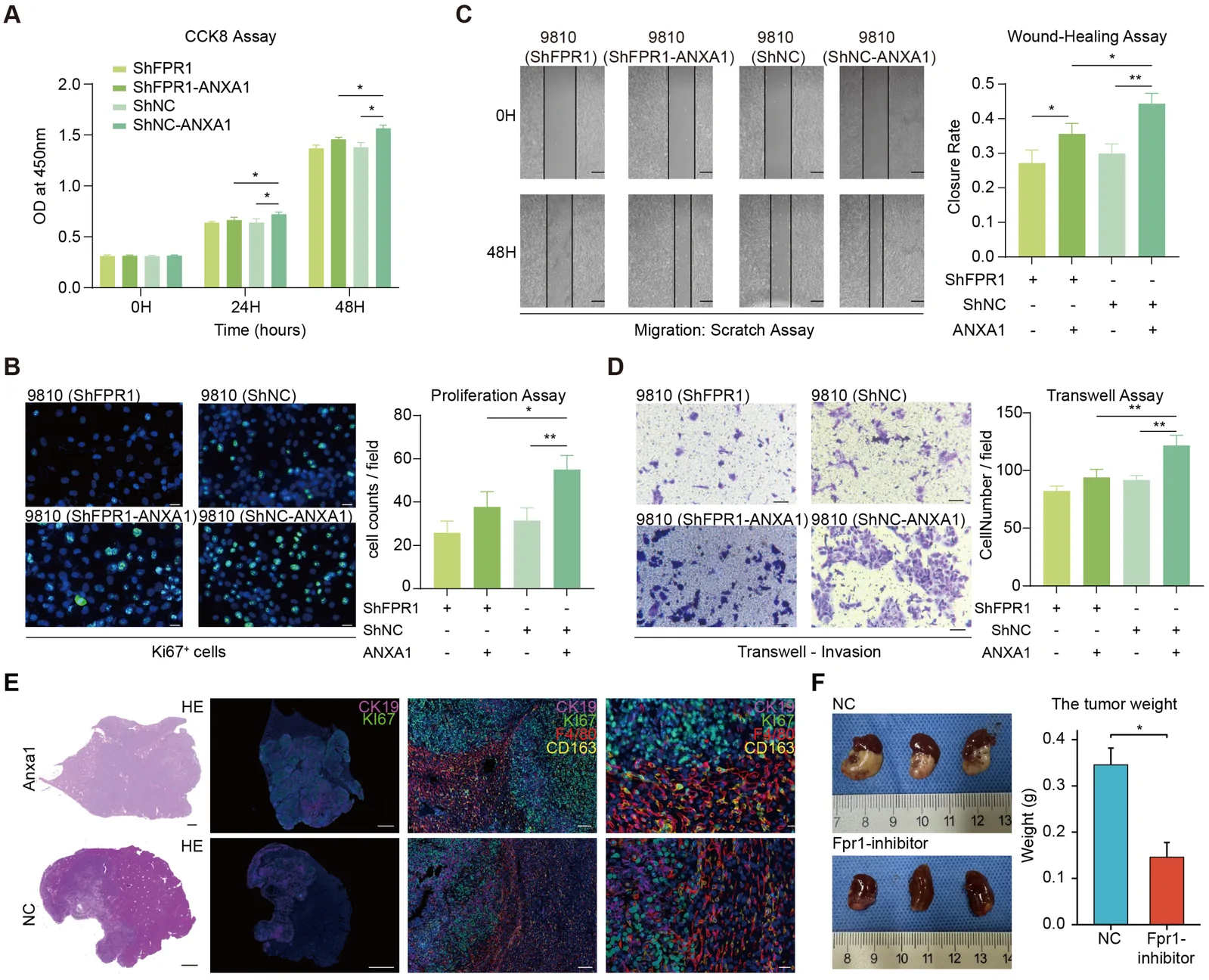
The progression of tumor was promoted by macrophages stimulated by ANXA1 protein. **(A)** Proliferation ability of HCCC-9810 in control groups and treated groups via CCK8 assay (24H, ShNC-ANXA1 vs ShNC, p < 0.05, ShNC-ANXA1 vs ShFPR1-ANXA1, p < 0.05; 48H, ShNC-ANXA1 vs ShNC, p < 0.05, ShNC-ANXA1 vs ShFPR1-ANXA1, p < 0.05). **(B)** Quantitative analysis of Ki67^+^ cells (Scale: 20μm; ShNC-ANXA1 vs ShNC, p < 0.01, ShNC-ANXA1 vs ShFPR1-ANXA1, p < 0.05). **(C and D)** Migration and invasion ability of HCCC-9810 in control groups and treated groups via scratch-wound healing assay and transwell invasion assay (scratch-wound healing assay, Scale: 100μm; ShNC-ANXA1 vs ShNC, p < 0.01, ShNC-ANXA1 vs ShFPR1-ANXA1, p < 0.05; transwell invasion assay, Scale: 50μm; ShNC-ANXA1 vs ShNC, p < 0.01, ShNC-ANXA1 vs ShFPR1-ANXA1, p < 0.01). **(E)** The IHC and mIF of M_2_-type macrophages and tumor (murine models, n = 10; HE, Scale: 1000μm; mIF, Left Scale: 2000μm; Middle Scale: 100μm; Right Scale: 20μm). **(F)** The tumor weight in NC and FPR1-inhibitor groups, respectively (murine models, n = 10; FPR1-inhibitor vs NC, p < 0.05). NC, negative control. * p < 0.05, ** p < 0.01, *** p < 0.001, **** p < 0.0001.

Macrophages treated with ANXA1 exhibited enhanced tumor cell proliferation as measured by the CCK8 assay. The proliferation effect reduced with decreasing FPR1 expression in macrophages (p < 0.05; Figure 7A). We observed the similar results by Ki67 staining of sections and quantification of Ki67^+^ cells (Figure 7B and S18B). Furthermore, we tested this phenomenon via EDU assay and obtained the same result (Figure S18A and C). The motility and invasive ability of tumor cells was found enhanced by the ANXA1-treated macrophages (motility, p < 0.01, Figure 7C and S18D; invasion, p < 0.01, Figure 7D). However, both impacts could be diminished by decreased FPR1 expression in macrophages (motility, p < 0.05, Figure 7B; invasion, p < 0.01, Figure 7D). Furthermore, tumor size was found to be indirectly associated with the ANXA1-FPR1 signaling pathway, as indicated by *in vivo* experiments (Figure 7E and F). The counts of M2-type macrophages in the tumor margin zone were regulated by the ANXA1-FPR1 signaling pathway (Figure 7E).

Overall, our results suggested that enriched CD4^+^ ANXA1^+^ T_cm_ cells in the tumor margin recruited and polarized macrophages via the ANXA1-FPR1 signaling axis, thereby indirectly promoting the progression of iCCA (Figure S19).

## Discussion

The tumor margin was considered a complex and active region^24^. The invasion of tumor cells was not limited to the dense tumor core, but also occurred at the margin of the tumor^25^. The immune microenvironment and the tumor-marginal immune signature in iCCA have not yet been investigated. In this study, we innovatively explored the immune microenvironment at the tumor margin through CyTOF analysis. Our primary finding is that a specific subset of CD4^+^ central memory T cells (T_cm_) significantly enriched in the margin area, and this enrichment is negatively associated with patient prognosis. Then, we elucidated the mechanisms underlying immune cell enrichment at the tumor margin and characterized active crosstalk between immune cells and tumor cells using bulk RNA-seq, spatial transcriptomics, and scRNA-seq, thereby facilitating the development of therapies for iCCA.

Cross-talk between tumor cells and immune cells, and interactions among immune cells themselves are critical for regulating tumor growth, invasion, and metastasis 26. In the related hepatocellular carcinoma, polarized tumor-associated macrophages inhibited the infiltration of CD8^+^ T cells, creating an immunosuppressive and cancer-promoting tumor microenvironment^27^. Multiple studies reported that the degree of CD4^+^ T_cm_ cell enrichment was negatively associated with patient prognosis 28. However, the specific role of CD4^+^ T_cm_ cells in cancer progression and the mechanisms underlying their enrichment remain elusive. Based on analyses of bulk RNA-seq, spatial transcriptomics, scRNA-seq data, qRT-PCR, and Western Blotting, we found that fibroblasts could recruit CD4^+^ T_cm_ cells via CCL19-CCR7 signaling pathway in the margin area. The HIF1α-FOSL2-ANXA1 signaling pathway could regulate ANXA1 expression in CD4^+^ T_cm_ cells.

The T cell activation-related genes (CD69 and CD28) were infrequently expressed in CD4^+^ ANXA1^+^ T_cm_ in the tumor margin zone, and the dysfunction and naïve scores of CD4^+^ ANXA1^+^ T_cm_ were high in all CD4^+^ T cell subsets. Combined with the CIBERSORT analysis, CD4^+^ ANXA1^+^ T_cm_ cells in the tumor margin zone was resting. The interaction between CD4^+^ ANXA1^+^ T_cm_ cells and macrophages was observed by analyzing the scRNA-seq data. Furthermore, we discovered that the ANXA1-FPR1 signaling pathway played a critical role in macrophage recruitment and polarization. However, the current study revealed that ANXA1 was essential in multiple cellular functions in cancer, including the infiltration of tumor-associated macrophages, the inhibitory function of Treg cells and the proliferation and metastasis of the tumor cells^29^, ^30^. In iCCA, this ANXA1-FPR1 signaling axis was not extensively studied before. Additionally, we found that ANXA1-stimulated macrophages could enhance the proliferation, migration and invasion of tumor cells^31^-^33^.

However, there were still several limitations should be acknowledged. Firstly, although we demonstrated the transcriptional regulation of ANXA1 by the HIF1α-FOSL2 complex, direct binding of this complex to the ANXA1 promoter was not validated via chromatin immunoprecipitation (ChIP) assays; thus, further ChIP experiments are warranted to definitively confirm this regulatory mechanism. Secondly, despite the use of mouse models to evaluate tumor progression, these models cannot fully recapitulate the complexity of the human tumor microenvironment or clinical scenarios.

In conclusion, our findings suggested that iCCA patients with a high enrichment of CD4^+^ T_cm_ cells at the tumor margin can have a worse prognosis. CD4^+^ T_cm_ cells at the tumor margin highly express ANXA1 and can be in a resting state. CD4^+^ ANXA1^+^ T_cm_ cells could mediate the polarization and recruitment of macrophages by activating the ANXA1-FPR1 axis in the margin zone of iCCA. Finally, ANXA1-stimulated macrophages promote tumor progression. Nevertheless, certain studies have explored the role and clinical significance of CD4^+^ ANXA1^+^ T_cm_ cells in iCCA. We here provided a novel prognostic marker, specifically the concentration of CD4^+^ ANXA1^+^ T_cm_ cells at the tumor margin. A recent study also demonstrated that therapeutic antibodies targeting ANXA1 could effectively inhibit cancer cell growth *in vivo* in breast cancer models^34^. Furthermore, therapies targeting ANXA1 have been reported as an effective approach against tumor progression. Thus, we hypothesized that CD4^+^ ANXA1^+^ T_cm_ cells were as a potential therapeutic target for iCCA.

## Supplementary Material

Supplementary materials and methods, figures and table 2.

Supplementary table 1.

Supplementary table 3.
