# Supplementary material for: Margin-enriched CD4+ central-memory T cells affect macrophages via ANXA1-FPR1 signaling axis to promote tumor progression in intrahepatic cholangiocarcinoma
Source: Int J Biol Sci. 2026 Aug 11;22(13):7225–39. doi: 10.7150/ijbs.136469 (PMC13540695; doi:10.7150/ijbs.136469)
Supplement: Supplementary file 1 [file ijbsv22p7225s1.pdf]

Materials and Methods

Patients and samples

All patients diagnosed with iCCA and undergoing surgical resection at Zhongshan Hospital, Fudan University between November 2012 and August 2019 were enrolled. None of the patients had received radiotherapy, chemotherapy or immunotherapy before surgery. The diagnosis of iCCA was confirmed pathologically. Fresh tumor tissues (at least 2 cm from the tumor margin), paratumor tissues (at least 2 cm from the tumor margin), and margin area tissues (1 cm-wide zones centered on the tumor margin) were obtained from 10 patients during surgical resection. These samples were placed into a tube for further CyTOF analysis (Table S1). Samples (including paratumor, tumor and the tumor margin) from 45 patients with iCCA were fixed in formalin and then embedded into paraffin (Table S2). This study was approved by the Research Ethics Committee of Zhongshan Hospital with patients' informed consent. Informed consent was taken from patients participated in this study for the clinical information and their samples.

CyTOF analysis of immune cells

CyTOF analysis was performed by PLTTech Inc. (Hangzhou, China) as described in the previous protocol[1]. 30 samples including tumor, paratumor and margin tissues from 10 patients were processed for the CyTOF analysis. A customized panel of 40 markers that encompassed a broad range of immune lineages was used. The conjugated antibodies were purchased directly from the supplier (Table S3). Leukocytes were washed and stained for viability with cisplatin and incubated with metal-conjugated surface-membrane antibodies for 30 min at 37°C. After that, the cells were fixed with fix perm buffer. Finally, cell intercalation (mixture of fixed perm buffer and iridium) was added for cellular fixation and visualization, and this procedure was allowed to proceed overnight before analysis on a Helios mass cytometer (Fludigm, USA). EQ Four Element Calibration Beads were used to normalize the signal. At least 1×10^5 cell events were collected for each sample. Files (.fcs) were uploaded into Cytobank, and populations of interest were manually gated. For further analysis, a random sampling of 100,000 cells from each fcs file was performed using the CyTOF kit program on R. Visualizations based on t-SNE and

clustering based on the FlowSOM/Renograph algorithms were then performed on these cells[2].

Single-cell RNA-seq analysis

Gene expression matrices were read and converted by the Seurat R package[3, 4]. Cells with less than 500 unique molecular identifiers (UMIs) or 25% mitochondrial UMI were excluded. The total cellular UMI counts were normalized by log transformation, and then we detected top 2000 highly variable features, performed PCA, clustering at resolution 1.5, t-Distributed Stochastic Neighbor Embedding (t-SNE) and Uniform Manifold Approximation and Projection (UMAP) analysis. Furthermore, we used feature plot, heatmap, and violin plot to visualize the gene expression in every cluster. The specific markers of each cluster (Wilcoxon Test; $log_2FC>0.25$; min.pct>0.25) were calculated by the FindAllMarkers function. Cell type for each cluster was annotated using the R package SingleR[5]. To infer statistically active TFs and their targets, the SCENIC was used[6]. The developmental potential of cell subsets was predicted by CytoTRACE[7]. Pseudotime analysis was performed using R package Monocle 3[8]. we manual validated the cell subtypes using established marker genes. We applied KEGG, GO, GSEA and ssGSEA analysis of $CD4^+$ $ANXA1^+$ T_{cm} to further analyze the role of differential expression. We analyzed the ligand-receptor pairs among the targeted cell subtypes to explore the potential cell-cell communication by the R package CellPhoneDB, and these pairs were visualized via the R package ggplot2[9]. InferCNV was used to identify somatic large-scale chromosomal copy number alterations using single-cell gene expression data.

10X Spatial transcriptomics data processing

In brief, The SCTranform function was used for data normalization, followed by PCA and UMAP for dimension reduction, and clustering was conducted with the default resolution of the first 30 PCs. The gene expression features were visualized using the SpatialFeaturePlot function, and signature scoring for $FPR1^+$ macrophage-like (CD68,

MACRO, FPR1, C1QA, C1QB, C1QC) and $CD4^+$ $ANXA1^+$ T_{cm}-like (CD4, CCR7, SELL, LEF1, TCF7, ANXA1) was performed with the AddModuleScore function[10].

Identification of the cell types based on Stereo-seq

To define the cell types in Stereo-seq data, we employed a marker-based scoring approach. Differentially expressed genes (DEGs) of cell types in the scRNA-seq data were identified using the FindAllMarkers function (min.pct = 0.1, p_val_adj < 0.05, avg_log2FC > log2(1.5)), and then ranked within each cell type according to p_val_adj. For $CD4^+$ $ANXA1^+$ T_{cm} cells and macrophages, we selected the top 50 DEGs for each cell type from scRNA-seq data and calculated module scores for each bin using the AddModuleScore function, with bins scoring in the top 1% designated as $CD4^+$ $ANXA1^+$ T_{cm}-enriched or macrophage-enriched. For $CCL19^+$ fibroblasts, we first identified bins with detectable CCL19 expression (expression > 0), then scored these CCL19-positive bins using the top 50 DEGs of fibroblasts, ultimately classifying bins in the top 2.5% module scores as $CCL19^+$ fibroblast-enriched bins.

Definition of Cellular Neighborhoods

The $CD4^+$ $ANXA1^+$ T_{cm}-enriched or macrophage-enriched bins were first identified. Subsequently, for each of these bins, we defined their respective cellular neighborhoods as all bins located within a radius of ≤1.5 (including the eight immediately adjacent bins) from the central bin of interest.

Spatial cross-correlation analysis

We calculated the level of the distribution of $FPR1^+$ macrophage (CD68, MACRO, FPR1, C1QA, C1QB, C1QC) and $FPR3^+$ macrophage (CD68, MACRO, FPR3, C1QA, C1QB, C1QC) in $CD4^+$ $ANXA1^+$ T_{cm} enriched bins (called as “$CD4^+$ $ANXA1^+$ T_{cm} neighborhood”) and other neighborhoods. We also calculated the feature genes of tumor cells in $FPR1^+$ macrophage enriched neighborhood and other neighborhoods. Then, the scoring for the hallmark of tumor cells in $FPR1^+$ macrophage enriched neighborhood and other neighborhoods was performed with the AddModuleScore function, respectively.

In order to exploring both cell types and tissue domains, BANKSY was performed to group the bins of sample into spatially informed clusters[11].

Bulk RNA-seq

Mouse Raw264.7 macrophage cell line was used for bulk RNA-seq. Raw264.7 was randomly divided into two groups: NC group (4 samples) and Ac2-26 group (Ac2-26, 200ng/ml, 37°C, for 4 hours; 5 samples). RNA was extracted using RNAiso Plus reagent and analyzed for quality using Agilent 2100 BioAnalyzer Expert (Agilent Technologies, Santa Clara, CA). Ribosomal RNA (rRNA) was depleted using a Ribo-zero Gold rRNA Removal kit (Illumina, San Diego, CA, USA). Strand-specific libraries were prepared using NEBNext Ultra II Directional RNA Library Prep Kit for Illumina (Ipswich, MA, USA). The libraries were sequenced on Illumina HiSeq X Ten to generate 2 × 150–base pair (bp) paired-end reads. Reads were aligned to mouse GRC38/mm10 genome with HISAT2 (v2.0.4). FPKMs (fragments per kilobase of transcript per million mapped reads) were calculated using the StringTie-eB software. Ensembl (GENCODE) transcripts were used for annotation.

The public data were from the National Genomics Data Center (NGDC) with accession number OEP000321.

Differential gene expression, gene ontology (GO) enrichment analyses, Kyoto Encyclopedia of Genes and Genomes (KEGG) analysis, the immune cell infiltration analysis, and gene set enrichment analysis (GSEA) were performed with RStudio (R version 4.0.2). CIBERSORT analysis was also used to deconvolute bulk RNA-seq profiles into cell-type scores.

Immunohistochemistry staining (IHC)

Immunohistochemistry was performed as standard procedures. In brief, the Paraffin-embedded iCCA sections were dewaxed in xylene three times and rehydrated by 100%, 90%, and 70% alcohol successively. Then, they were pretreated with citrate buffer (pH 6.0) for antigen retrieval. Sections were pretreated with hydrogen peroxide in PBS and then washed with tris-buffered saline (TBS) for two times, followed by blocking with 3%

bovine serum albumin (BSA)/ TBS. Sections were incubated with primary antibody: anti-CD4 (Servicebio, GB13064-1), anti-CD3 (Servicebio, GB11014). To evaluate the staining index, three zones with a width of 500 μm were selected from tumor areas, areas around the margin (the zone centered on the margin), and paratumor tissues.

Immunofluorescence staining (IF)

Briefly, the Paraffin-embedded iCCA sections were deparaffined by xylene and rehydrated by 100%, 90%, 70% alcohol, respectively. Slides were washed by PBS for two times and added in the blocking buffer. Sections were incubated with the following antibody: anti-CCR7 (Abcam, ab253187), anti-CD4 (Maxim, RMA-0620), anti-CD68 (Maxim, kit0026), anti-Annexin A1/ANXA1 (Abcam, ab214486), anti-FPR1 (Abcam, ab113531), anti-CD45RO (Abcam, ab216024), anti-CD3 (Abcam, ab135372), anti-mouse CK19 (Abcam, ab52625), anti-Ki67 (Abcam, ab15580), anti-mouse F4/80 (Abcam, ab6640), anti-mouse CD163 (Abcam, ab182422), anti-mouse ARG1 (Abcam, ab96183), anti-mouse CD4 (Abcam, ab288724), anti-mouse ANXA1(Abcam, ab214486), anti-mouse CCR7 (Affinity Biosciences, AF5293), anti-mouse CD62L (Abcam, ab119834), anti-mouse CD44 (Affinity Biosciences, DF6392), anti-FPR1 (Bioss, bs-2572R), anti-CD206 (Abcam, ab64693), anti-ARG1 (Abcam, ab96183), anti-CD69 (Abcam, ab233396) and anti-a-SMA (Cell Signaling, 19245). The cell nuclei were stained with DAPI (Sigma). Next, sections were incubated by corresponding secondary antibody: Alexa Fluor™ 555 Tyramide Reagent (Thermo Fisher Scientific, B40955), Alexa Fluor™ 647 Tyramide Reagent (Thermo Fisher Scientific, B40958), Donkey anti-Rabbit IgG (H+L) Highly Cross-Adsorbed Secondary Antibody, Alexa Fluor™ 488 (A-21206). Images were obtained with a fluorescent microscope. Next, sections were incubated by corresponding secondary antibody. For each slide, three zones with a width of 200 μm and length of 250 μm from tumor tissues, paratumor tissues, and the areas centered on the margin were selected for image capture and further analysis. We quantified the target cell count in the selected squares across different regions and calculated the average number of cells per region. For each slide, five randomly selected fields (200 μm × 250 μm) adjacent to these zones were analyzed, and the number of $CD4^+$ T_{cm} cells within each field was counted. The

mean CD4$^+$ T_{cm} number was calculated per patient. We classified samples with target cell counts in tumor margin ranking in the top 50% as high-enrichment patients, and the rest as low-enrichment patients.

Flow cytometry and CD4$^+$ T_{cm} isolation

The tumor, margin and paratumor tissues were isolated, minced and resuspended into the Tumor Dissociation Kit (Miltenyi Biotec) diluted into DMEM. The tissues were dissociated with gentleMACS Octo Dissociator and then pass through 70-um strainer to the single-cell suspension following the manufacturer's recommendations. The samples were centrifuged at 400 × g for 10 minutes without braking and resuspended in PBS. Cell suspensions were performed at the indicated antibody dilutions according to the manufacturer's recommendations for 30 min at 4 °C and protected from light. In the case of intracellular staining, cell suspensions were incubated with Fixation/Permeabilization Kit (BD Pharmingen, Cat #554714) and then performed at the indicated intracellular targeted antibody dilutions according to the manufacturer's recommendations shielded from light for 30 min at 4 °C. The stained cells were washed twice and fixed with BD cytofix (20 minutes, 4°C, and protect from light). Antibodies were as follow: PerCP/Cyanine5.5 anti-human CD3 Antibody (Biolegend, Cat #317336), APC anti-human CD4 Antibody (Biolegend, Cat #300514), Zombie NIR™ Fixable Viability Kit (Biolegend, Cat #423105), Brilliant Violet 421™ anti-human CD197 (CCR7) Antibody (Biolegend, Cat #353208), PE anti-human CD45 Antibody (Biolegend, Cat #304008), PerCP/Cyanine5.5 anti-human CD68 Antibody (Biolegend, Cat #333814), PE/Cyanine7 anti-human CD86 Antibody (Biolegend, Cat #374210), Brilliant Violet 605 anti-human CD45RO (Biolegend, Cat #304238) APC anti-human CD206 (MMR) Antibody (Biolegend, Cat #321110), FITC anti-human CD69 (Biolegend, Cat #310904), PE anti-mouse CD163 Antibody (Biolegend, Cat #155307) and FITC anti-mouse F4/80 Recombinant Antibody (Biolegend, Cat #157309). Flow cytometry data were captured on a LSRFortessa X-20(BD Biosciences) and analyzed using FlowJo version 7.6.1 (Tree Star). The targeted cells were flow-sorted into complete 1640 medium after gating on

$CD4^+$ T_{cm}, M_1-type macrophage, M_2-type macrophage and $CD4^+$ $CD69^+$ T_{cm}. All flow sorting experiments were performed on a FACsAria cytometer (BD Biosciences).

$CD4^+$ T_n cells isolation and $CD4^+$ Th1 Differentiation

PBMCs were isolated from peripheral blood using Ficoll density-gradient centrifugation. naïve $CD4^+$ T cells were isolated and purified using naïve $CD4^+$ T cell isolation kit (Miltenyi Biotec, Bergisch Gladbach, Germany) according to the manufacturer's instructions[12].

The naïve $CD4^+$ T cells were stimulated with human CD3/CD28 T cell activator (STEMCELL Technologies) and human Th1 differentiation supplement (STEMCELL Technologies), and were cultured in Roswell Park Memorial Institute (RPMI) 1640 medium. On day 3, differentiated Th1 cells are ready for use[13].

qRT-PCR

qRT-PCR experiments were performed as described previously[14]. Total RNA was extracted by using Trizol (Invitrogen), and the quantity and quality of RNA was determined via the Thermofisher NanoDrop™ reader (ND - 1000). The M-MLV Reverse Transcriptase kit (Thermo Fisher Scientific) was used to perform the reverse transcription reaction according to the protocol. TaqMan fast advanced master mix (Thermo Fisher Scientific) was used for real-time PCR.

Western blotting analysis

Western blot was performed as previously described[15]. Briefly, total protein lysates were run in Nupage 4–12% Bis-Tris gradient precast gels (Invitrogen) in MOPS buffer. Proteins were subjected to long migration time (~2.5h) in ice to allow correct separation and visualization of FPR1. Primary antibodies used in this study are: anti-FPR1 (Bioss, bs-2572R), anti-FOSL2 (Proteintech, 15832-1-AP), anti-HIF1α (BOSTER, A00013-1), anti-β-ACTIN (Abcam, ab8227), anti-GAPDH (Abcam, ab9485), anti-CCL19 (Abcam, ab192877), anti-ANXA1 (Proteintech, 21990-1-AP), anti-mouse FOSL2 (Invitrogen, PA5-99459), anti-mouse HIF1α (Abcam, ab308433), anti-mouse β-ACTIN (Abcam, ab8226).

Isolation, culture and characterization of bone marrow–derived macrophage (BMDM)

Murine bone marrow–derived macrophages (BMDM) were isolated and activated as previously described[16]. Briefly, bone marrow cells from femur of C57BL/6 male mice were isolated and cultured for 7 days in DMEM: F12 (Gibco, Life technologies, USA) supplemented with 10% FBS (Gibco, USA) and 50 ng/ml M-CSF (R&D Systems, USA). Media was changed every 3 days and contaminating nonadherent cells are eliminated and adherent cells are harvested for further stimulated. BMDM was identified by their co-expression of F4/80 and Dapi via IF staining.

Cell culture

Human iCCA cell lines (HCCC-9810 and HuCCT1, ATCC) were incubated in DMEM medium (high glucose, 10% FBS, 100 U/ml penicillin and streptomycin). The human monocytic cell line (THP-1, ATCC) was incubated in complete 1640 medium (10% FBS, 100 U/ml penicillin and streptomycin). The mouse macrophage line Raw264.7 was incubated in complete 1640 medium (10% FBS, 100 U/ml penicillin and streptomycin). The mouse iCCA cell line (SHYK0002) was incubated in complete 1640 medium (10% FBS, 100 U/ml penicillin and streptomycin). All cells were grown in collagen-coated plastic (50ug/mL) at 37ºC in a humidified 5% CO_2 atmosphere.

Lentivirus production and cell transduction

Lentivirus vectors of FPR1 knockdown was purchased from Genomeditech (Shanghai, China). The negative control was termed as shNC, and FPR1 knockdown lentiviruses was termed as shFPR1. Lentivirus transfection was conducted as previously described [17].

ANXA1 siRNA and nonsense siRNA was obtained from Thermo Fisher Scientific. $CD4^+$ T_{cm} cells were plated in 12 wells and were transiently transfected with 2 µg of siRNAs plus 4 µl Lipofectamine 3000 according to the manufacturer’s instruction. 36 h later, cells were harvested. The cell lysates were blotted for specific antibodies against ANXA1 (Table S3).

Cell treatment

THP-1 cells were induced to be differentiated into macrophages by treating with phorbol myristate acetate (PMA) for 24 hours. Macrophages were incubated at each complete 1640 medium with 100ng/ml recombinant human ANXA1 (R&D Systems, Minneapolis, MN), Ac2-26 (MedChemExpress, HY-P1098) or HCH6-1 (MedChemExpress, HY-101283). The cell culture supernatants of macrophages described above were collected. Raw264.7 were incubated at each complete 1640 medium with Ac2-26 (MedChemExpress, HY-P1098; 200ng/ml) or HCH6-1 (MedChemExpress, HY-101283; 50μM).

BMDM were incubated at each complete 1640 medium with Ac2-26 (MedChemExpress, HY-P1098; 200ng/ml) or HCH6-1 (MedChemExpress, HY-101283; 50μM). The cell culture supernatants of macrophages described above were collected.

$CD4^+$ T_{cm} cells were cultured in T cell media, and part of them were treated with hypoxia.

HCCC-9810/HuCCT1 were allocated in four groups (macrophage groups, macrophage-ANXA1 groups, ShFPR1-ANXA1 groups and the ShNC-ANXA1 groups).

In the macrophage groups, cells were incubated in DMEM medium (high glucose, 10% FBS, 100 U/ml penicillin, and 100 U/ml streptomycin) with the macrophage culture supernatants. In the HCCC-9810/HuCCT1 (macrophage-ANXA1) groups, cells were incubated in DMEM medium with the ANXA1-stimulated macrophage culture supernatants. In the HCCC-9810/HuCCT1 (ShFPR1-ANXA1) groups, cells were incubated in DMEM medium with the ANXA1-stimulated macrophage (knocked down FPR1 groups) culture supernatants. In the HCCC-9810/HuCCT1 (ShNC-ANXA1) groups, cells were incubated in DMEM medium with the ANXA1-stimulated macrophage (NC groups) culture supernatants.

Metabolism assays

Each group ($CD4^+$ T_{cm}, $CD4^+$ T_n, $CD4^+$ Th1) was plated in quintuplicate in the Seahorse XF24 Cell Culture Microplates (Agilent) (1×106 per well) with 500 μl Seahorse XF base medium (Agilent), 100 mM sodium pyruvate (11360070, Gibco) and 2.5 M glucose (A2494001, Gibco) for OCR measurements, and with 500 μl Seahorse XF base medium

with 100 mM sodium pyruvate and 2 mM glutamine (25030081, Gibco) for ECAR measurements for 1 hr in the absence of CO_2 at 37°C prior to the start of the metabolism assay. OCR and ECAR were assessed as previously described

Transwell migration assay

Macrophages were placed on the inner well. Complete 1640 medium with or without the protein ANXA1 was added in the outer well. After 24 hours incubation, migrated cells were fixed with 4% formaldehyde and stained using crystal violet (KeyGEN BioTECH, Cat NO: KGA229).

Wound-healing assay

When the HCCC-9810/HuCCT1 reached about 90%confluence in culture plate wells, medium was replaced with serum-free medium for 24 hours. Then, wounds were made with a 200μL - pipette tip at the bottom of the wells.

Cell invasion assay

Cells were suspended in serum-free medium and seeded on the inner well pre-coated with Matrigel Matrix (BD Biosciences, USA). DMEM medium (high glucose, 10% FBS) with or without the macrophage-culture supernatants was added in the outer well. After 48 hours incubation, cells were fixed with 4% formaldehyde and stained using crystal violet (KeyGEN BioTECH, Cat NO: KGA229).

CCK8 assay

In brief, cells were seeded in 96-well plates (2×10^3 cells/well) with the treatment as described. CCK8 reagent was added to each well for 2 hours at 37°C, and the OD value was measured at 450nm.

EDU assay

Cells were seeded on gelatin-fibronectin-coated cover slips and fixed in 4% paraformaldehyde for 15 minutes at room temperature. The Click-iT EdU Alexa Fluor®

594 Imaging Kit (Invitrogen) was used to detect EdU. Nuclei were stained with DAPI (Sigma).

Ki-67 positive cells assay

Cells were seeded on gelatin-fibronectin-coated cover slips and fixed in 4% paraformaldehyde for 15 minutes at room temperature. Proliferation was examined by immunofluorescent staining for Ki-67 according to the manufacturers' instructions (Abcam).

Enzyme-linked immunosorbent assay (ELISA)

The levels of ANXA1 secreted by $CD4^+$ T_{cm} cells isolated from the tumor margin and adjacent non-tumor liver tissues of the iCCA patients were quantified by Human ANXA1 ELISA kits (MyBioSource). THP-1 cells were transduced with scramble or shFPR1 lentivirus for 48 h and primed with 100ng/ml PMA for 24h as above. Then, cells were stimulated by 100ng/ml recombinant human ANXA1. The culture medium supernatant was obtained and stored for the next step. Human IL-6 ELISA kits, human VEGF ELISA kits, Human IL-10 ELISA kits and Human TGF-β ELISA kits (BioLegend) were used to quantify the levels of relative proteins in the supernatant. BMDM was divided into three groups (NC group, Ac2-26 treated group, Ac2-26+HCH6-1 group). The cell culture supernatants of these groups were collected. Mouse Il-10 ELISA kits, mouse Vegf ELISA kits and mouse Tgf-β ELISA kits (BioLegend) were used to quantify the levels of relative proteins in the supernatant.

In vivo experiment

Animal model

Animal experiments were performed under a project license (NO. 2019-185) granted by the institutional ethics board of Zhongshan Hospital, Fudan University, in compliance with institutional guidelines for the care and use of animals. To avoid gender- or age-related effects, male C57 mice of 8-week-old (20-25g) were used.

Mouse tumor-bearing model and treatment

Mice were anesthetized, and orthotopic tumors were established by sub-capsular inoculation of 5×10^5 iCCA tumor cell line (SHYK0002) into the left liver lobe by laparotomy.

CCL19 group and CCL19 plus anti-CCR7 group: Mice were weighted twice weekly and sacrificed at day 12. For chemokine treatment, 5 μg CCL19 (BioLegend, 587806) diluted in 20 μL PBS were injected into the tumor margin, while the control group received an equivalent volume of PBS injection on day 6 after tumor engraftment. For CCR7 blocking, mouse CCR7 antibody (R&D Systems, clone no. 4B12) was injected intravenously at 10 μg/mouse at day 6 after tumor engraftment as described[18, 19].

NC group and ANXA1 group: 100ng/ml ANXA1 (Novus Biologicals, Littleton, USA, NBP2-52001) was injected into the tumor margin, while the control group received an equivalent volume of PBS injection on day 6 after tumor engraftment.

NC group and FPR1-inhibitor group: 50μM HCH6-1 (MedChemExpress, HY-101283) was injected into the tumor margin, while the control group received an equivalent volume of PBS injection on day 6 after tumor engraftment.

Mouse $CD4^+$ T cell isolation and culture

Briefly, Single cell suspensions were prepared from spleens and peripheral lymph nodes after red blood cell lysis (Sigma-Aldrich). $CD4^+$ T cells were enriched by magnetic cell sorting using the mouse $CD4^+$ T cell isolation kit II (Miltenyi Biotec) in accordance with manufacturer's instructions[20].

Data availabilty

The CyTOF data can be obtained through contacting the corresponding author. The Stereo-seq data and the scRNA-seq data were from the China National GeneBank (CNGB) Sequence Archive (CNSA, accession code: CNP0002199). The 10X ST data was from the research (http://lifeome.net/supp/livercancer-st/data.htm).

Statistical analysis

Statistical analysis was performed with R script (version 4.0.2) and GraphPad Prism 7.0 (SanDiego, CA) software. Kaplan-Meier method was used for survival analysis. Unpaired two-sided Wilcoxon rank-sum test, paired two-sided Wilcoxon rank-sum test, Tukey's multiple comparisons test and $\chi 2$ test were used for comparisons. Adjust p value or p value < 0.05 was considered statistically significant. Abbreviations for p values are as follows: $p < 0.05$ = ∗, $p < 0.01$ = ∗∗, $p < 0.001$ = ∗∗∗, $p < 0.0001$ = ∗∗∗∗; with only significant p values shown.

Reference

1. Han G, Spitzer M, Bendall S, Fantl W, Nolan G. Metal-isotope-tagged monoclonal antibodies for high-dimensional mass cytometry. Nature protocols. 2018;13(10):2121–48.

2. Lim C, Lee Y, Pan L, Lai L, Chua C, Wasser M, et al. Multidimensional analyses reveal distinct immune microenvironment in hepatitis B virus-related hepatocellular carcinoma. Gut. 2019;68(5):916–27.

3. Butler A, Hoffman P, Smibert P, Papalexi E, Satija R. Integrating single-cell transcriptomic data across different conditions, technologies, and species. Nature biotechnology. 2018;36(5):411–20.

4. Stuart T, Butler A, Hoffman P, Hafemeister C, Papalexi E, Mauck W, et al. Comprehensive Integration of Single-Cell Data. Cell. 2019;177(7):1888–902.e21.

5. Aran D, Looney A, Liu L, Wu E, Fong V, Hsu A, et al. Reference-based analysis of lung single-cell sequencing reveals a transitional profibrotic macrophage. Nature immunology. 2019;20(2):163–72.

6. Aibar S, González-Blas C, Moerman T, Huynh-Thu V, Imrichova H, Hulselmans G, et al. SCENIC: single-cell regulatory network inference and clustering. Nature methods. 2017;14(11):1083–6.

7. Chen L, Yandong G, Han Z, Hua Y, Yang Z, Zhilei B, et al. Delineating spatiotemporal and hierarchical development of human fetal innate lymphoid cells. Cell Res. 2021;31(10).

8. Trapnell C, Cacchiarelli D, Grimsby J, Pokharel P, Li S, Morse M, et al. The dynamics and regulators of cell fate decisions are revealed by pseudotemporal ordering of single cells. Nature biotechnology. 2014;32(4):381–6.

9. Vento-Tormo R, Efremova M, Botting R, Turco M, Vento-Tormo M, Meyer K, et al. Single-cell reconstruction of the early maternal-fetal interface in humans. Nature. 2018;563(7731):347–53.

10. Xiong A, Zhang J, Chen Y, Zhang Y, Yang F. Integrated single-cell transcriptomic analyses reveal that GPNMB-high macrophages promote PN-MES transition and impede T cell activation in GBM. EBioMedicine. 2022;83:104239.

11. Vipul S, Nigel C, Joseph L, Yifei Y, Jinyue L, Wan Kee C, et al. BANKSY unifies cell typing and tissue domain segmentation for scalable spatial omics data analysis. Nat Genet. 2024;56(3).

12. Linyu G, Xiaojun T, Kangxing Z, Dandan W, Shiying W, Genhong Y, et al. MicroRNA-663 induces immune dysregulation by inhibiting TGF-β1 production in bone marrow-derived mesenchymal stem cells in patients with systemic lupus erythematosus. Cell Mol Immunol. 2019;16(3).

13. Yunhong T, Chunshan L, Zhiyong L, Meiling A, Baiyao W, Kunpeng D, et al. Exosomal B7-H4 from irradiated glioblastoma cells contributes to increase FoxP3 expression of differentiating Th1 cells and promotes tumor growth. Redox Biol. 2022;56(0).

14. Garelli A, Heredia F, Casimiro A, Macedo A, Nunes C, Garcez M, et al. Dilp8 requires the neuronal relaxin receptor Lgr3 to couple growth to developmental timing. Nature communications. 2015;6:8732.

15. Zhang K, Yao E, Chuang E, Chen B, Chuang E, Chuang P. mTORC1 signaling facilitates differential stem cell differentiation to shape the developing murine lung and is associated with mitochondrial capacity. Nature communications. 2022;13(1):7252.

16. Xia Z, Ricardo G, David M M. The isolation and characterization of murine macrophages. Curr Protoc Immunol. 2008(0).

17. Yang C, Yuan W, Yang X, Li P, Wang J, Han J, et al. Circular RNA circ-ITCH inhibits bladder cancer progression by sponging miR-17/miR-224 and regulating p21, PTEN expression. Molecular cancer. 2018;17(1):19.

18. Zhang Y, Liu G, Zeng Q, Wu W, Lei K, Zhang C, et al. CCL19-producing fibroblasts promote tertiary lymphoid structure formation enhancing anti-tumor IgG response in colorectal cancer liver metastasis. Cancer cell. 2024;42(8):1370–85.e9.

19. Pei G, Yao Y, Yang Q, Wang M, Wang Y, Wu J, et al. Lymphangiogenesis in kidney and lymph node mediates renal inflammation and fibrosis. Science advances. 2019;5(6):eaaw5075.

20. Yu W, Sheng-Nan S, Qing L, Yang-Yang Y, Jian G, Kun W, et al. Autocrine Complement Inhibits IL10-Dependent T-cell-Mediated Antitumor Immunity to Promote Tumor Progression. Cancer Discov. 2016;6(9).

Supplement Figure

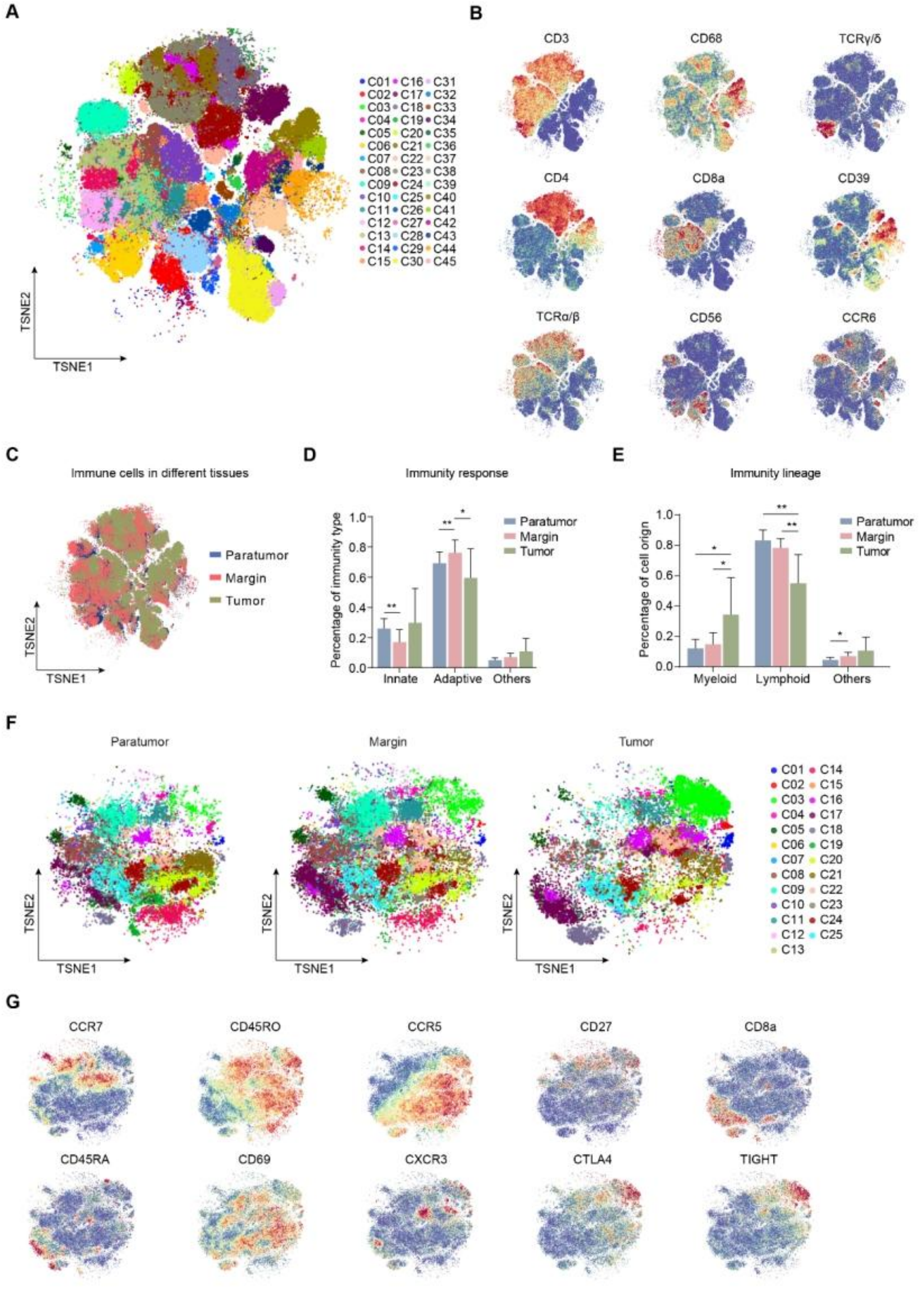

Figure S1

The characteristics of CD45⁺ cells and CD4⁺ T cell subsets based on the data of cytometry by time of flight (CYTOF)

(A) Overview of 45 immune cell clusters

(B) Marker genes for different immune cell subsets

(C) Overview of immune cell subsets in paratumor, margin and tumor, respectively.

(D) Percentage of different immunity types in paratumor, margin and tumor, respectively.

(E) Percentage of different immunity lineages in paratumor, margin and tumor, respectively.

(F) Overview of $CD4^+$ T cell in paratumor, margin and tumor, respectively.

(G) Marker genes for $CD4^+$ T cell subsets

* $p<0.05$, ** $p<0.01$, *** $p<0.001$, **** $p<0.0001$

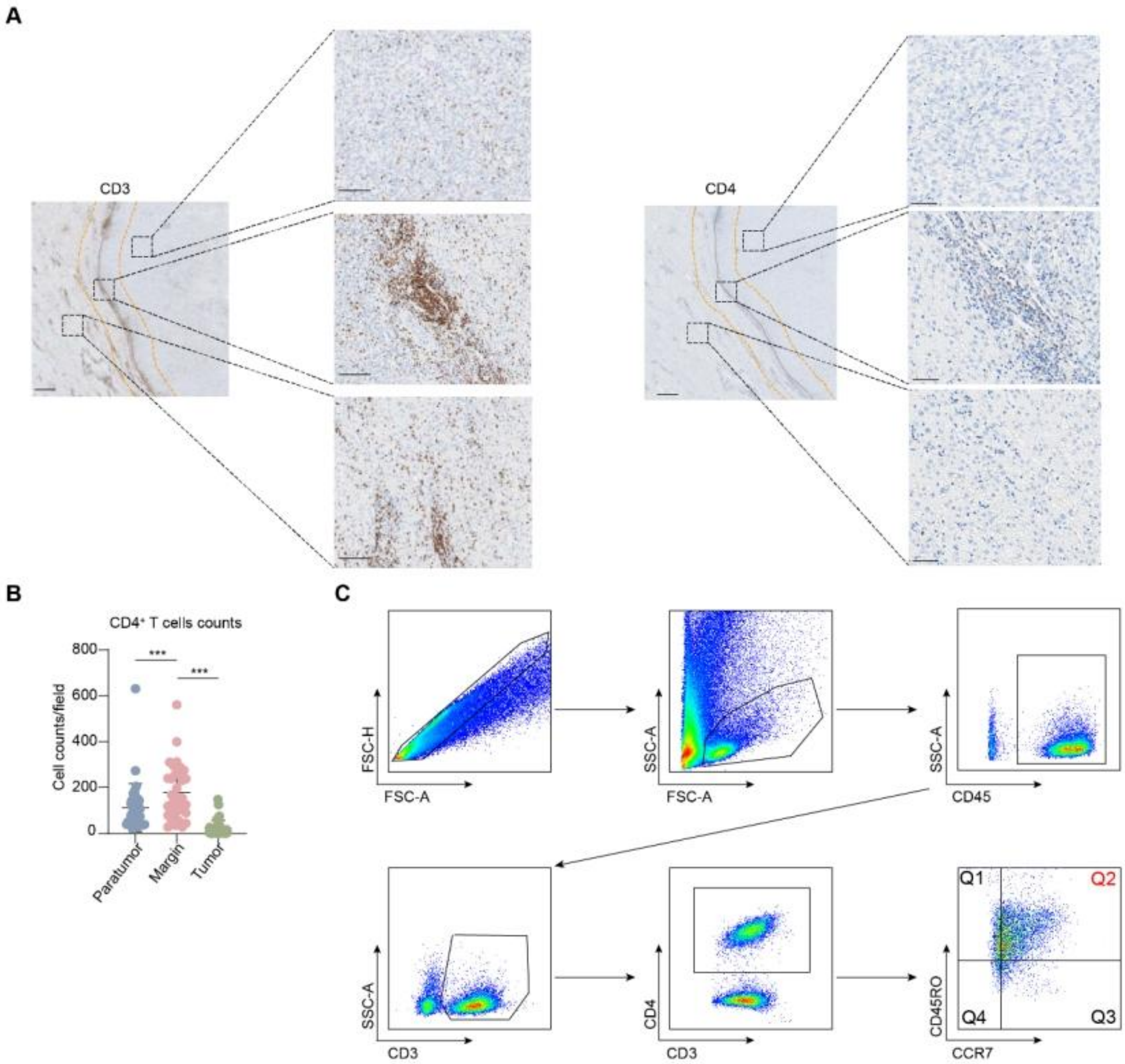

Figure S2

The clinical implications of accumulated CD4+ central-memory T cells (CD4+ T_{cm}) in the margin zone of intrahepatic cholangiocarcinoma (iCCA).

(A) Immunohistochemical staining for CD4+ T cells in the margin zone of iCCA (Right Scale: 500μm; Left Scale: 50μm).

(B) Accumulation of CD4+ T cells in the margin zone validated via IHC staining (Margin vs Paratumor, $p < 0.001$; Margin vs Tumor, $p < 0.001$).

(C) Flow cytometry plots of CD4+ T_{cm} cells in iCCA

* p<0.05, ** p<0.01, *** p<0.001, **** p<0.0001

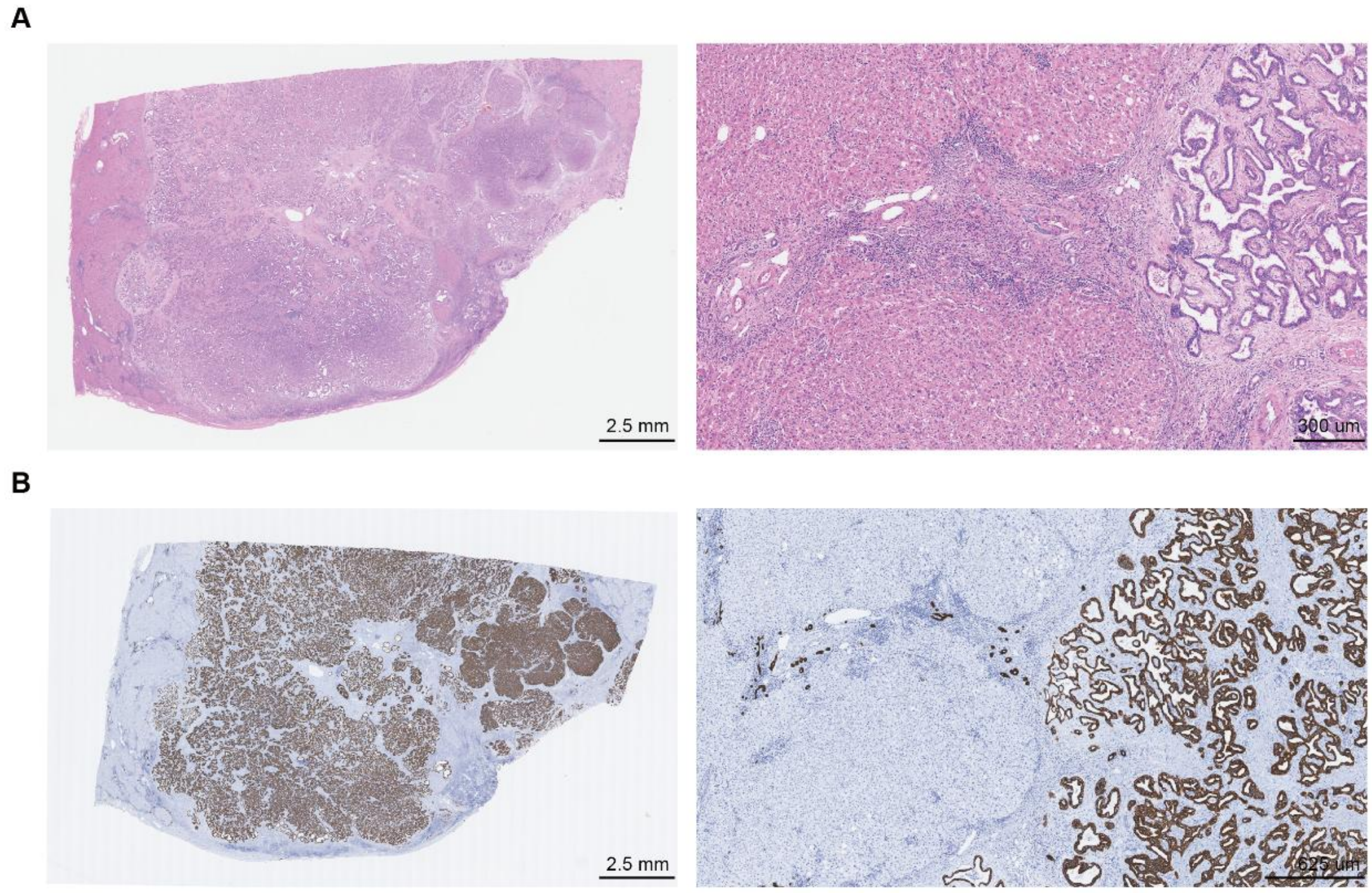

Figure S3. Liver pathological analysis and CK19 expression analysis in the liver and tumor tissues of iCCA patients.

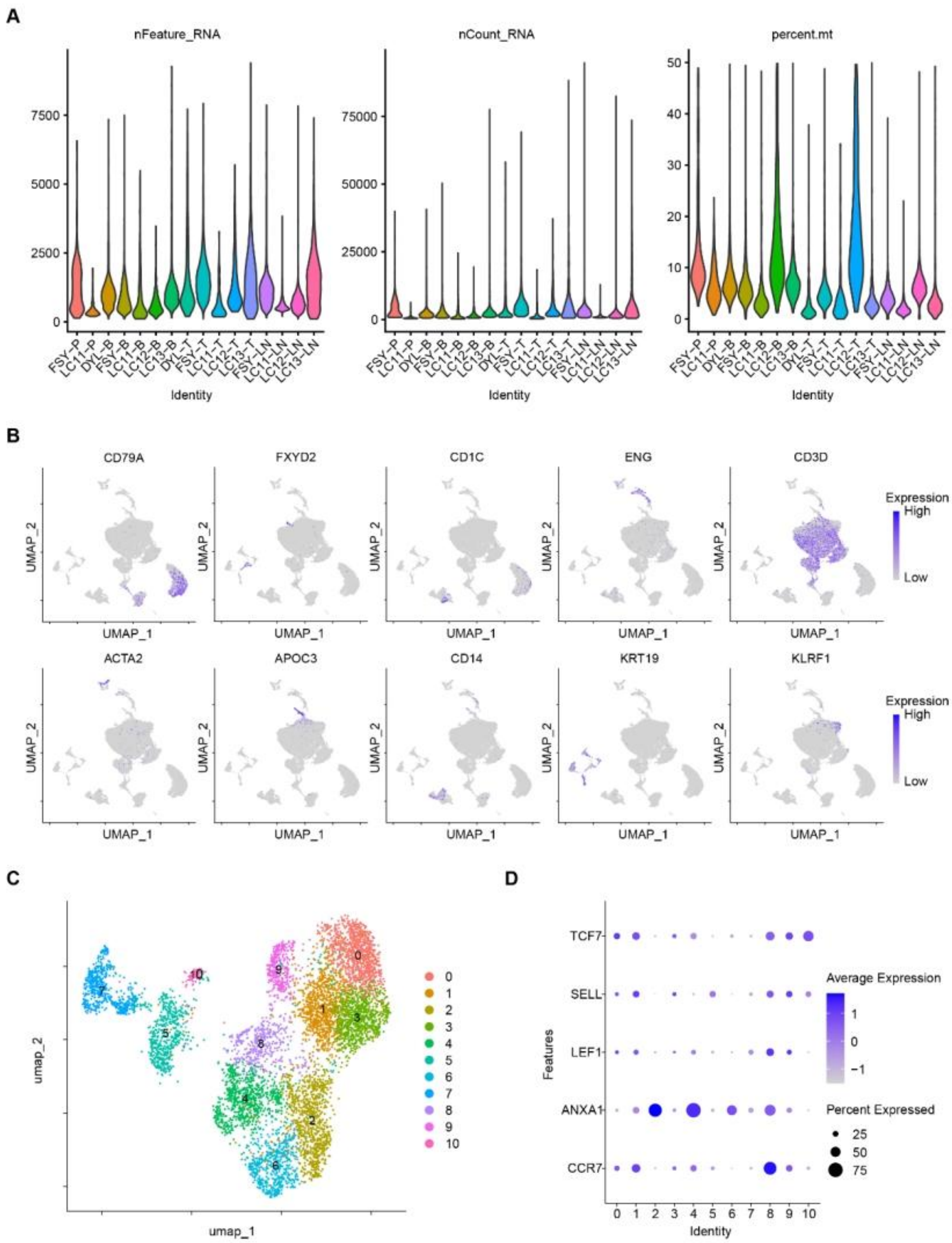

Figure S4

The characterizations of CD4⁺ ANXA1⁺ T_{cm} cells based on scRNA-seq.

(A) Violin plot showing gene number in each cell (nFeature), total count number of all genes in each cell (nCount), and percentage of mitochondrial gene number in total gene number in each cell (percent.mt).

(B) Marker genes of different cell types.

(C) UMAP plots of clusters in $CD4^+$ T cells.

(D) The expression of relative genes in different clusters.

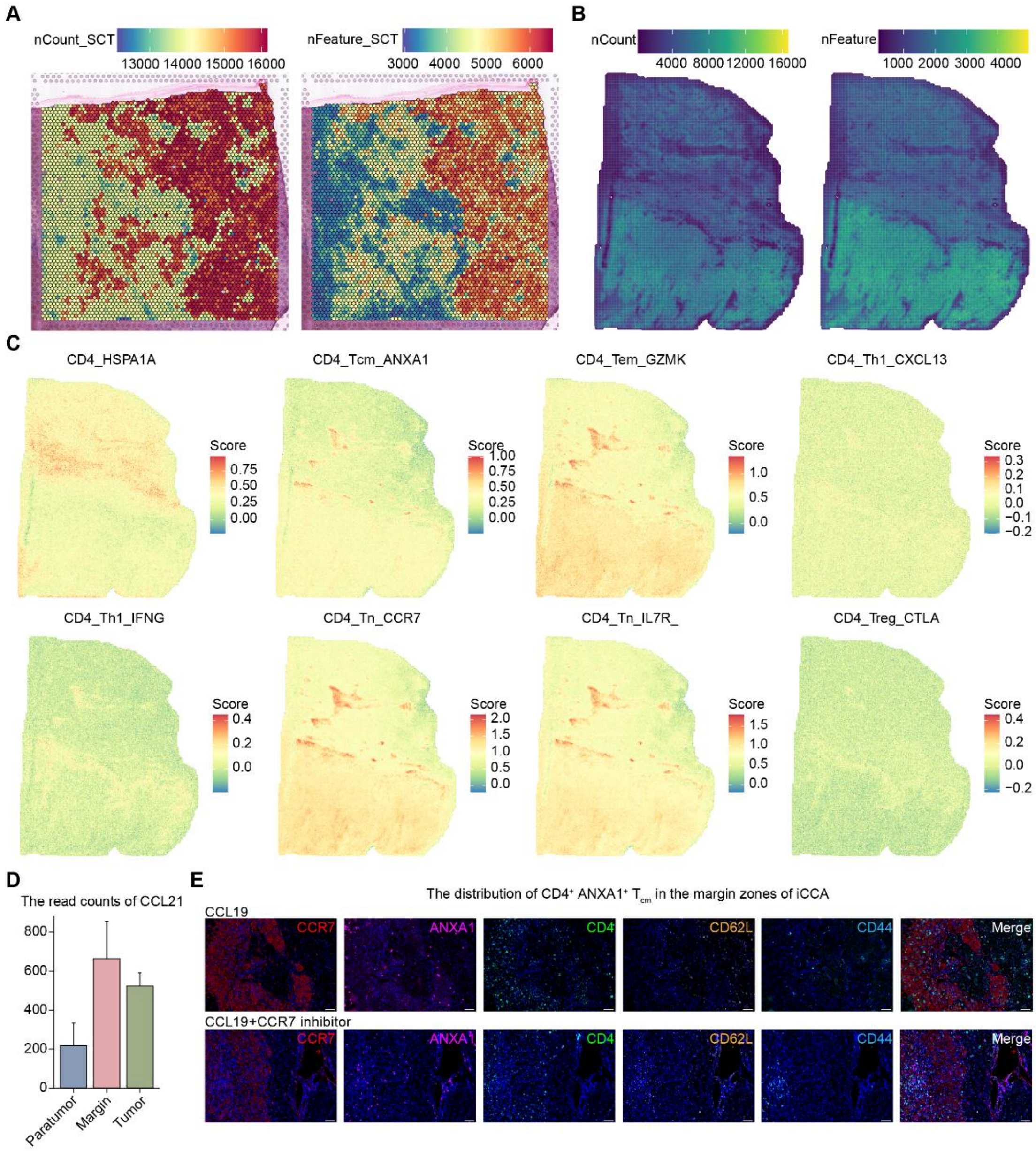

Figure S5

Overview of the spatial transcriptomic sections.

(A and B) Spatial feature plots analysis of nFeature and nCount in margin zones of iCCA.

(C) UMAP plots of clusters in $CD4^+$ T cells.

(D) The read counts of CCL21 in different zones of iCCA based on bulk RNA-seq.

(E) The distribution of $CD4^+$ $ANXA1^+$ T_{cm} in the margin zone of iCCA via mIF staining.

A

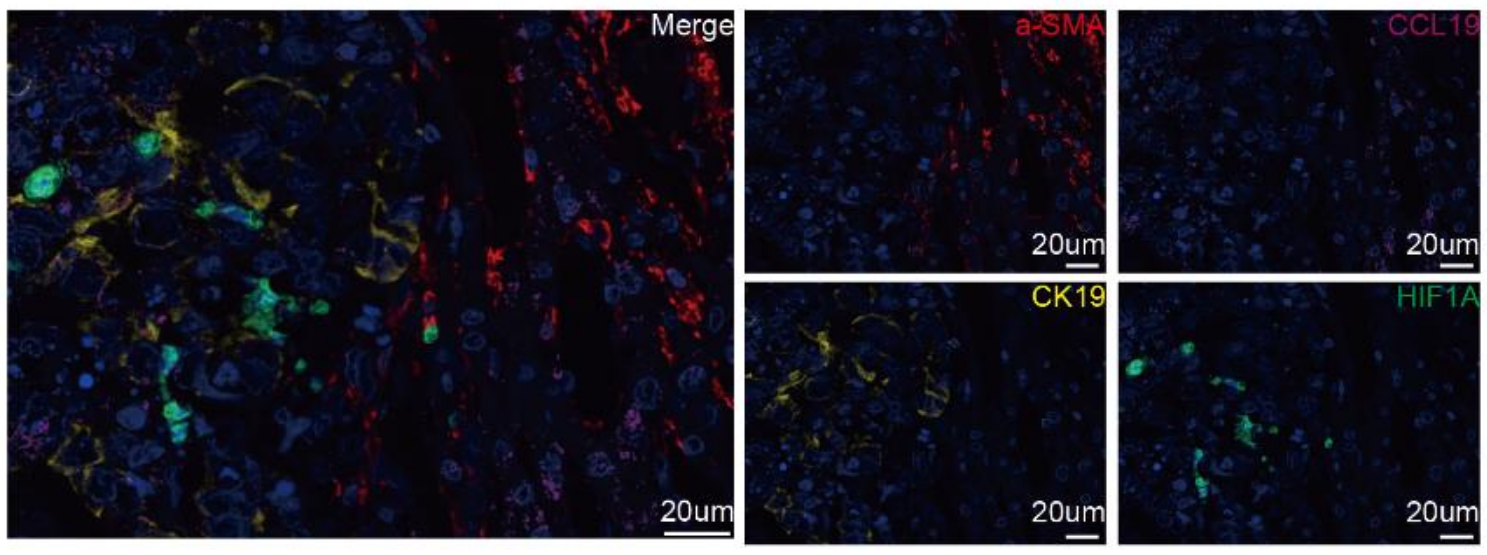

Figure S6

CCL19⁺ fibroblast and HIF1α⁺ cells in margin zone of iCCA.

(A) mIF staining showing CCL19⁺ fibroblast and HIF1α⁺ cells in tumor margin.

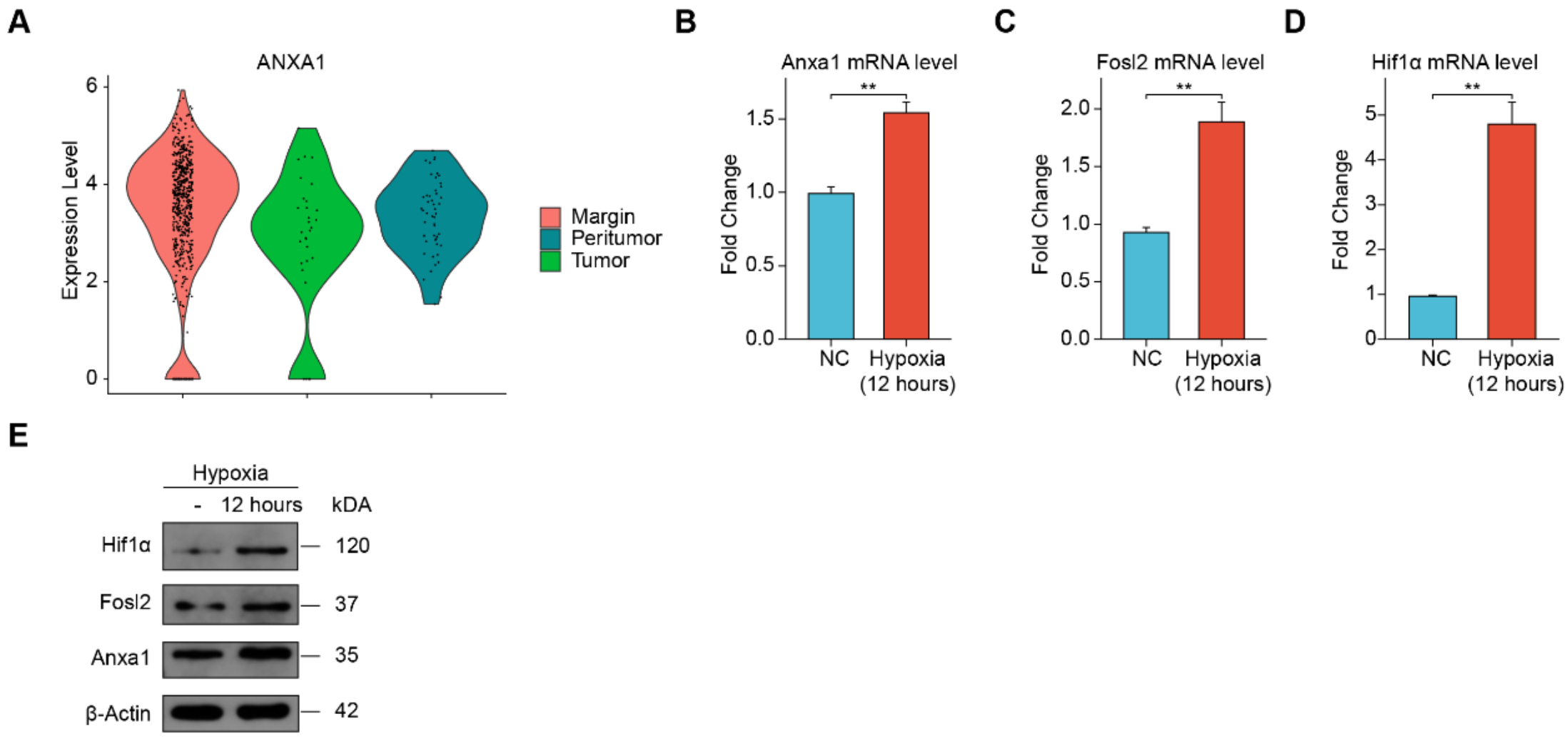

Figure S7

CD4⁺ ANXA1⁺ T_{cm} cells governed by HIF1α-FOSL2 signaling pathway in margin zone of iCCA.

(A) The mRNA level of ANXA1 in paratumor, margin and tumor, respectively.

(B) qRT-PCR analysis of the expression of Anxa1 in $CD4^+$ T_{cm} cells (Hypoxia vs NC, $p < 0.01$).

(C) qRT-PCR analysis of the expression of Fosl2 in $CD4^+$ T_{cm} cells (Hypoxia vs NC, $p < 0.01$).

(D) qRT-PCR analysis of the expression of Hif1α in $CD4^+$ T_{cm} cells (Hypoxia vs NC, $p < 0.01$).

(E) WB analysis of the expression of proteins involved in Hif1α-Fosl2-Anxa1 signaling pathway in $CD4^+$ T_{cm} cells.

NC, negative control

* $p<0.05$, ** $p<0.01$, *** $p<0.001$, **** $p<0.0001$

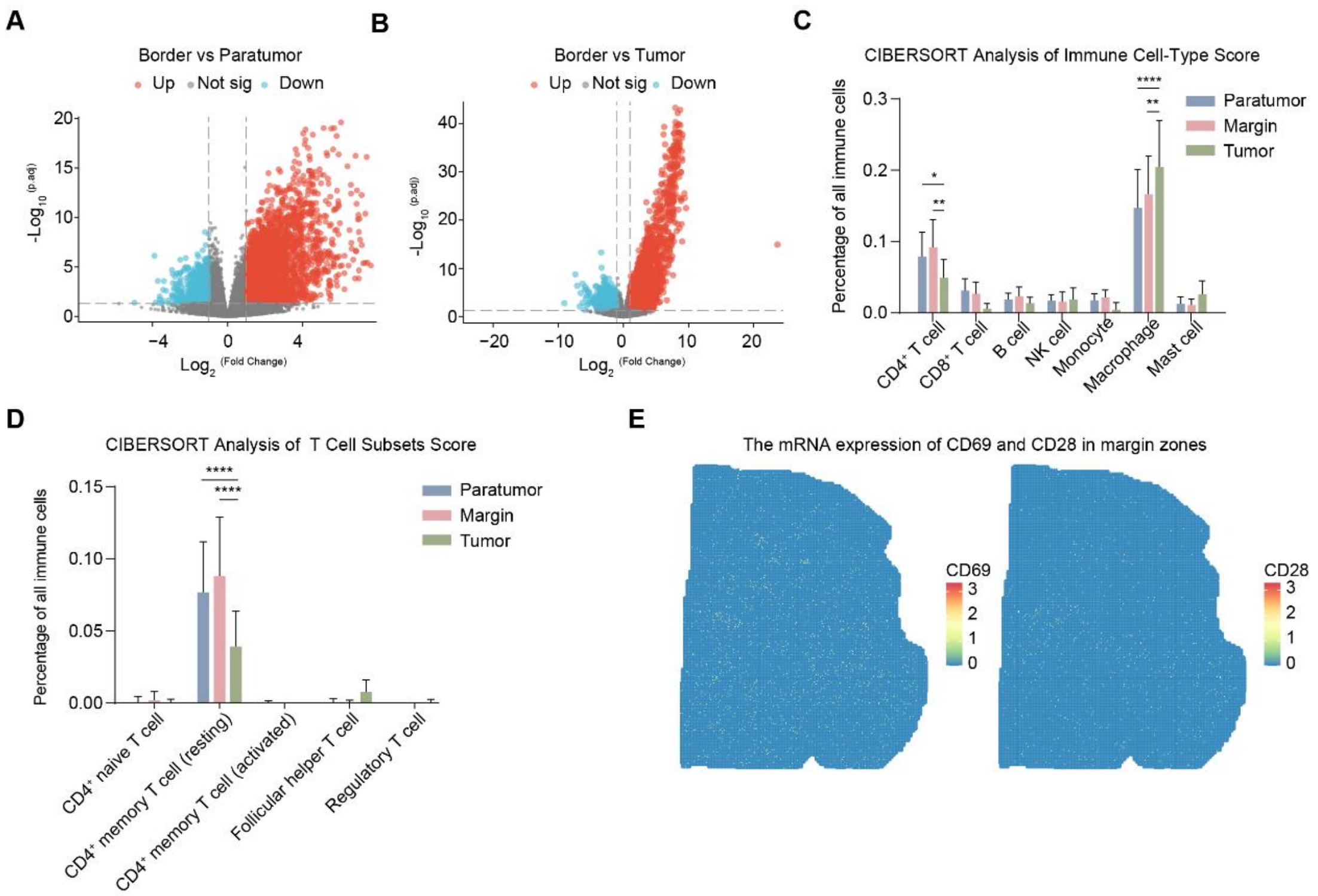

Figure S8

The gene characteristics and immune signatures of margin zones in iCCA based on bulk RNA-seq.

(A) Overview of different expressed genes between the margin zones and paratumor zones in iCCA.

(B) Overview of different expressed genes between the margin zones and tumor zones in iCCA.

(C) CIBERSORT analysis of immune cell subsets in paratumor, margin and tumor, respectively.

(D) CIBORSORT analysis of $CD4^+$ T cell subsets (Margin vs Tumor, $p < 0.0001$; Tumor vs Paratumor, $p < 0.0001$).

(E) The mRNA levels of CD69 and CD28 in margin zones of iCCA based ST.

* p<0.05, ** p<0.01, *** p<0.001, **** p<0.0001

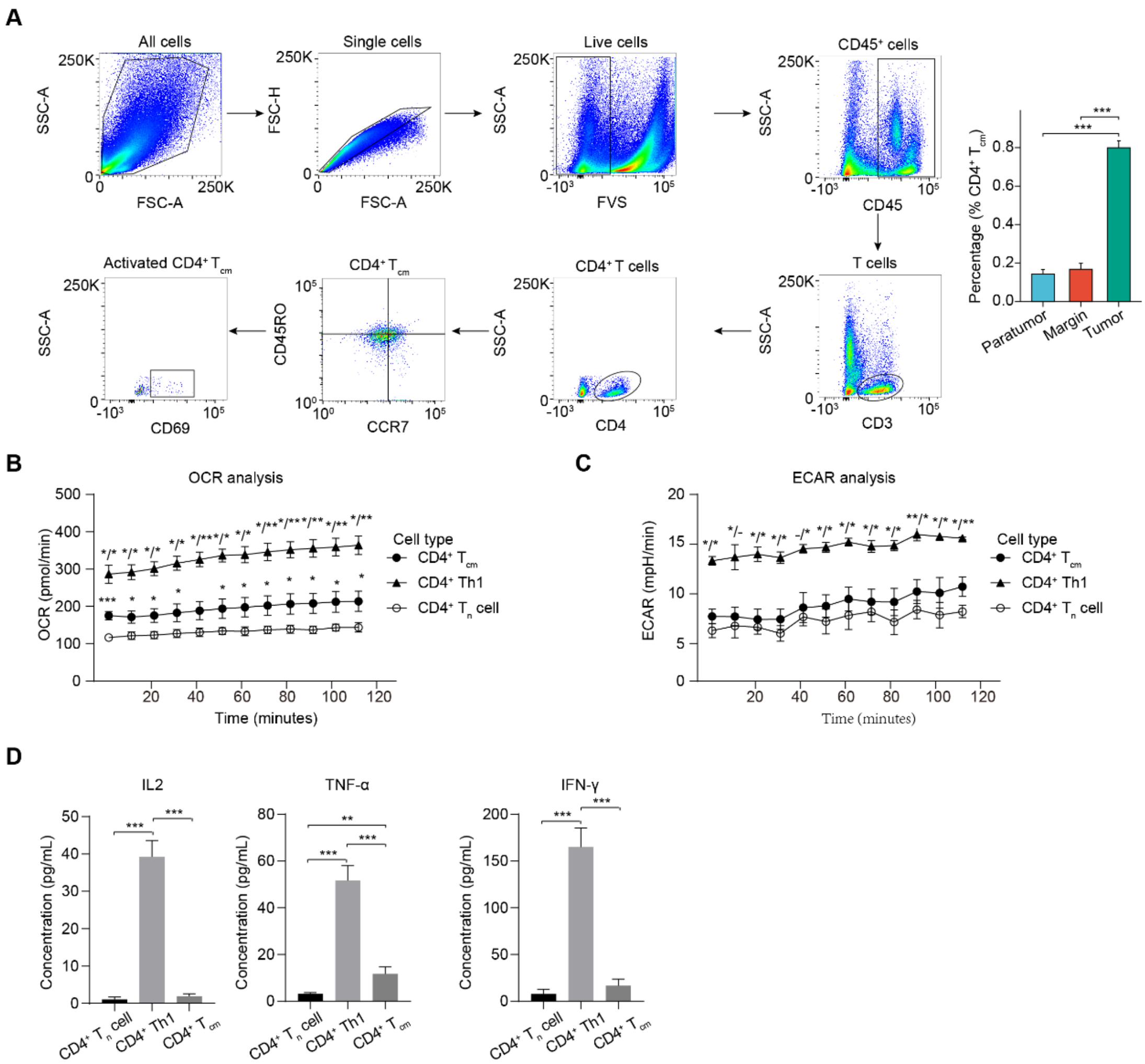

Figure S9

The state of CD4$^+$ ANXA1$^+$ T_{cm} cells in different zones of iCCA.

(A) The state of CD4$^+$ ANXA1$^+$ T_{cm} cells in iCCA based on flow cytometry (Margin vs Tumor, $p < 0.001$; Tumor vs Paratumor, $p < 0.001$).

(B and C) illustrate schematic diagrams of oxygen consumption rate (OCR) and ECAR time courses of CD4$^+$ T_{cm}, CD4$^+$ Th1 and CD4$^+$ T_n cells.

(D) Cytokine expression of IL2, TNF-α and IFN-γ secreted by CD4$^+$ T_{cm}, CD4$^+$ Th1 and CD4$^+$ T_n cells (IL-2, CD4$^+$ Th1 vs CD4$^+$ T_{cm}, $p < 0.001$, CD4$^+$ Th1 vs CD4$^+$ T_n, $p < 0.001$; TNF-a, CD4$^+$ Th1 vs CD4$^+$ T_{cm}, $p < 0.001$, CD4$^+$ Th1 vs CD4$^+$ T_n, $p < 0.001$, CD4$^+$ T_{cm} vs CD4$^+$ T_n, $p < 0.01$; IFN-γ, CD4$^+$ Th1 vs CD4$^+$ T_{cm}, $p < 0.001$, CD4$^+$ Th1 vs CD4$^+$ T_n, $p < 0.001$).

* p<0.05, ** p<0.01, *** p<0.001, **** p<0.0001

A

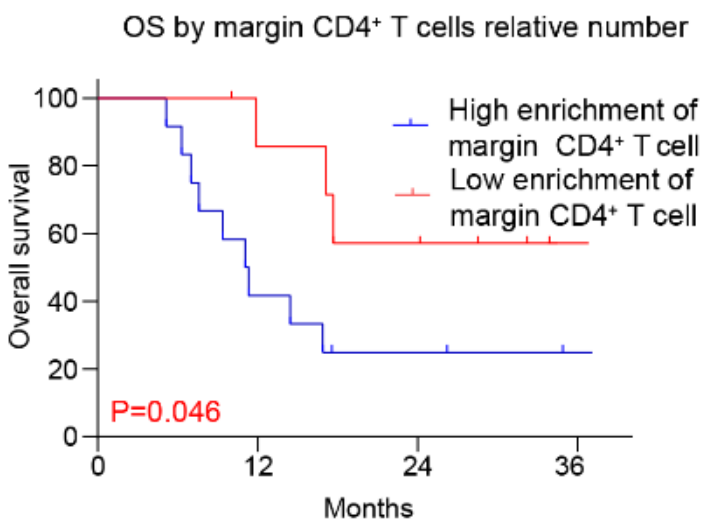

Figure S10

The clinical significance of $CD4^+$ T cells in the margin zones of iCCA.

(A) OS analysis of different enrichment of $CD4^+$ T_{cm} cell in the tumor margin in iCCA

(n=36; OS, $p = 0.046$).

OS, overall survival

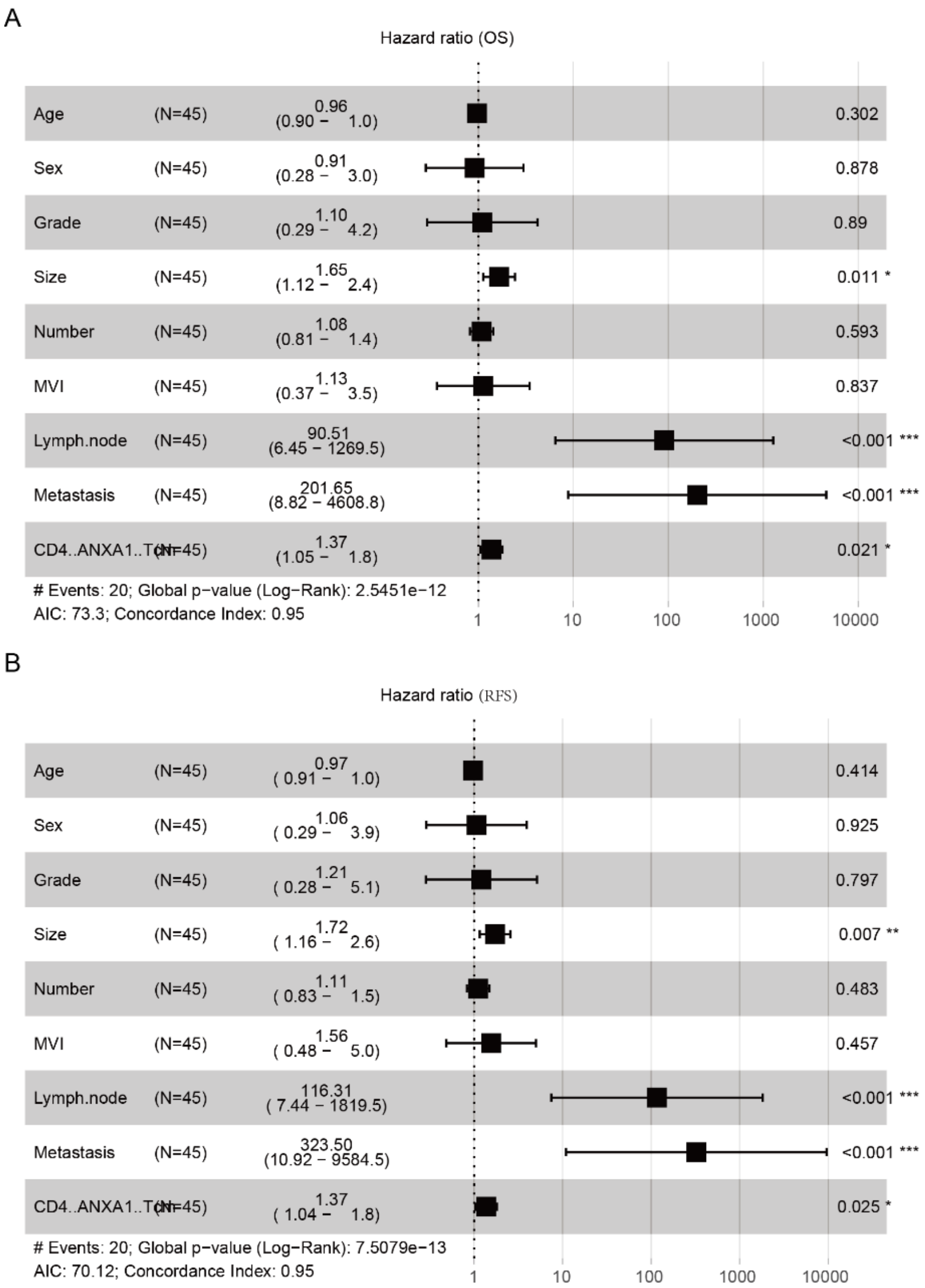

Figure S11

The forest plot of HR (95% CI) for OS and RFS of the iCCA patients with high or low

$CD4^{+}$ $ANXA1^{+}$ T_{cm} enrichment in tumor margin.

(A) Forest plot of hazard ratio of OS.

(B) Forest plot of hazard ratio of RFS.

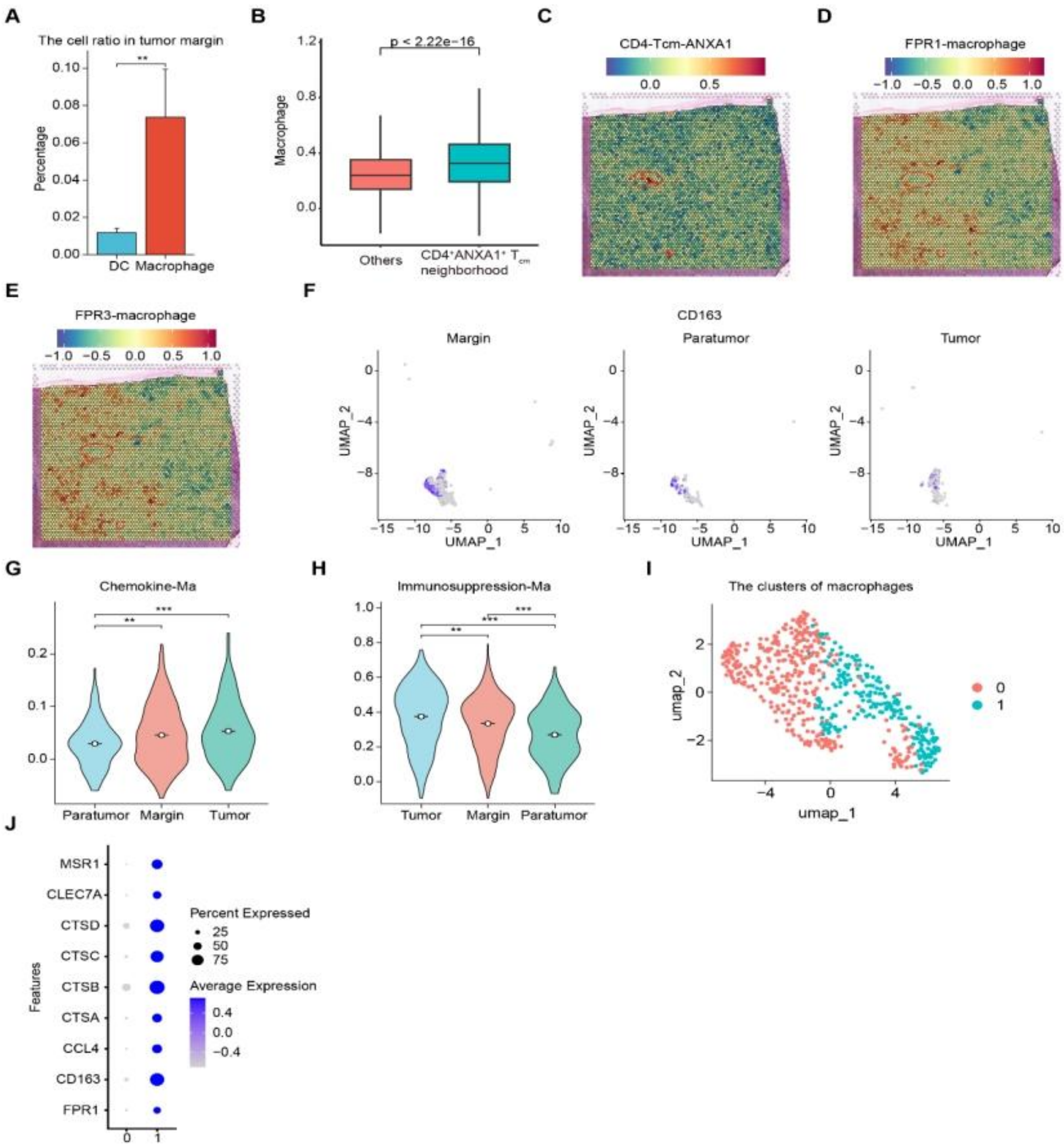

Figure S12

The characterizations of macrophages based on public scRNA-seq and ST database.

(A) Bar charts showing the proportion of macrophage and DC as a proportion of total cells (Macrophage vs DC, $p < 0.01$).

(B) The scores of macrophages in $CD4^+$ $ANXA1^+$ T_{cm} and other neighborhoods in margin zones of iCCA ($CD4^+$ $ANXA1^+$ T_{cm} neighborhood vs Others, $p < 2.22e-16$).

(C) The ssGSEA score of $CD4^+$ $ANXA1^+$ T_{cm} in margin zones of iCCA.

(D) The ssGSEA score of $FPR1^+$ macrophage in margin zones of iCCA.

(E) The ssGSEA score of $FPR1^+$ macrophage in margin zones of iCCA.

(F) Feature plots of CD163 mRNA level in macrophages in different zones of iCCA.

(G) ssGSEA analysis of chemokine in macrophage (Margin vs Paratumor, $p < 0.01$; Tumor vs Paratumor, $p < 0.001$).

(H) ssGSEA analysis of immunosuppressive in macrophage (Margin vs Paratumor, $p < 0.01$; Tumor vs Paratumor, $p < 0.001$; Margin vs Tumor, $p < 0.001$).

(I) The UMAP plot of different clusters of macrophages.

(J) The mRNA level of M2-type macrophage marker genes of clusters based on scRNA-seq

DC, dendritic cell; T_{cm}, central memory T cell;

* $p<0.05$, ** $p<0.01$, *** $p<0.001$, **** $p<0.0001$

A

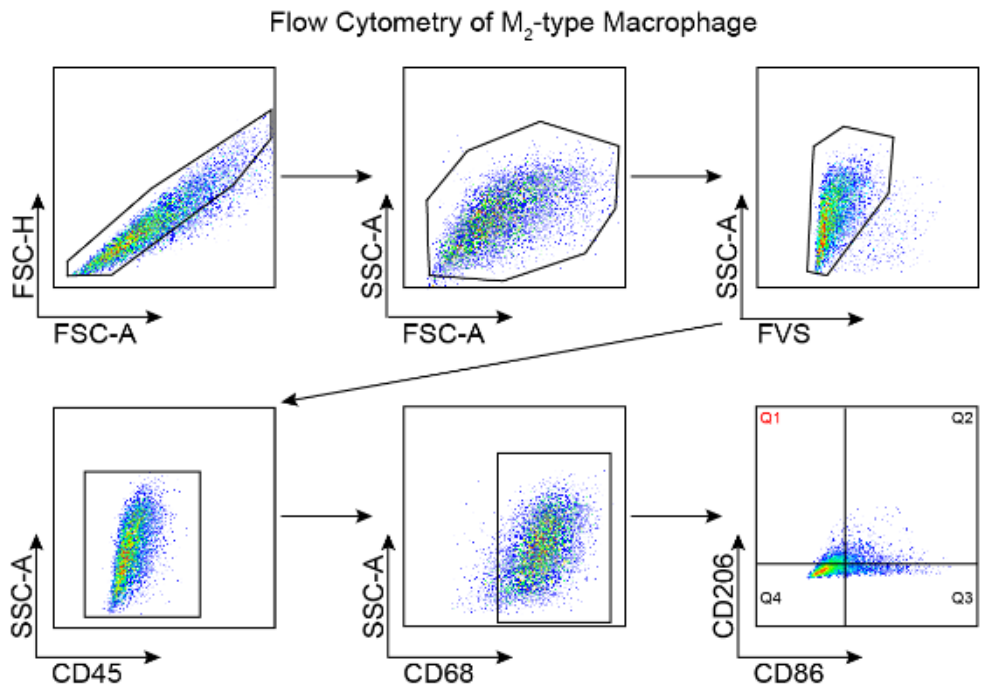

Figure S13

The flow cytometry of M_2-type macrophages.

(A) The flow cytometry of M_2-type macrophages.

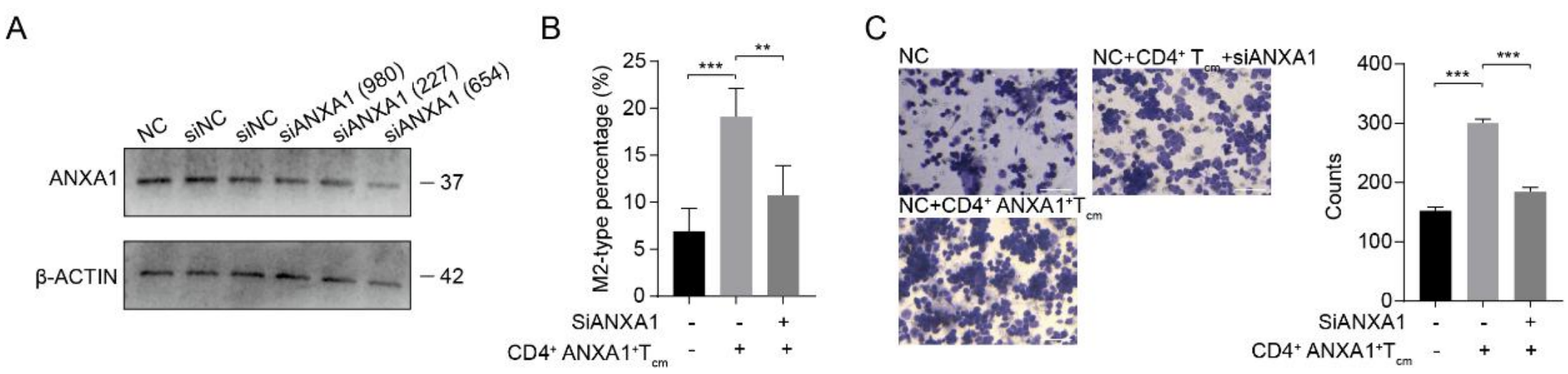

Figure S14

$CD4^+$ $ANXA1^+$ T_{cm} regulating macrophage recruitment and polarization via ANXA1.

(A) WB analysis showing the ANXA1 protein expression.

(B) The percentage of M2-type macrophages in total macrophages (NC + $CD4^+$ $ANXA1^+$ T_{cm} vs NC, $p < 0.001$; NC + $CD4^+$ $ANXA1^+$ T_{cm} vs NC + $CD4^+$ T_{cm} + siANXA1, $p < 0.01$).

(C) The migration ability of macrophages assessed by the transwell assay (Scale: 100μm).

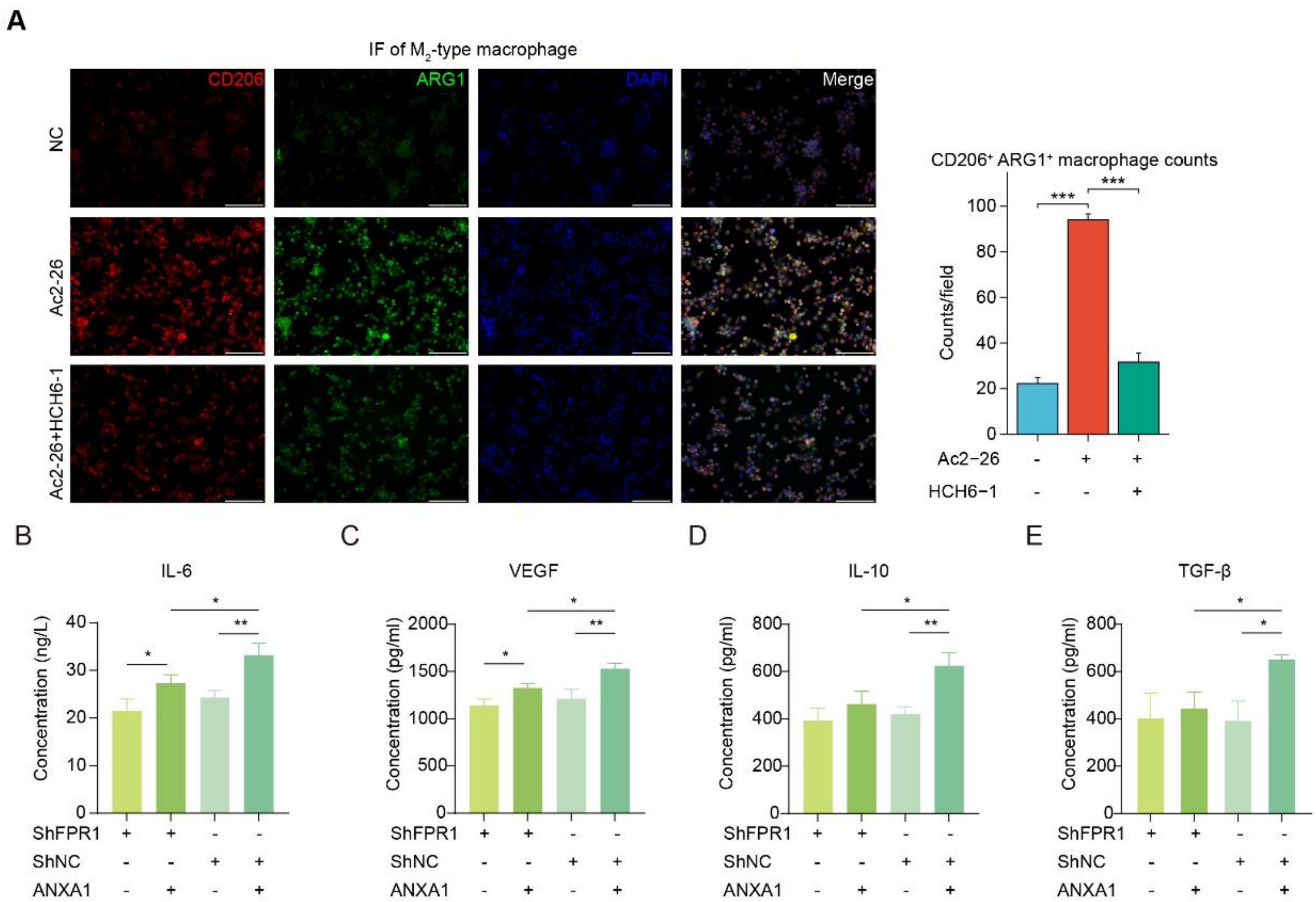

Figure S15

The polarization of macrophages regulated by $CD4^{+}$ $ANXA1^{+}$ T_{cm} via ANXA1-FPR1 signaling pathway.

(A) The mIF staining and counts of M_2-type macrophages (in vitro experiment, n = 10).

(B to E) Cytokine expression of IL-6, VEGF, IL-10 and TGF-β determined by ELISA.

NC, negative control

* p<0.05, ** p<0.01, *** p<0.001, **** p<0.0001

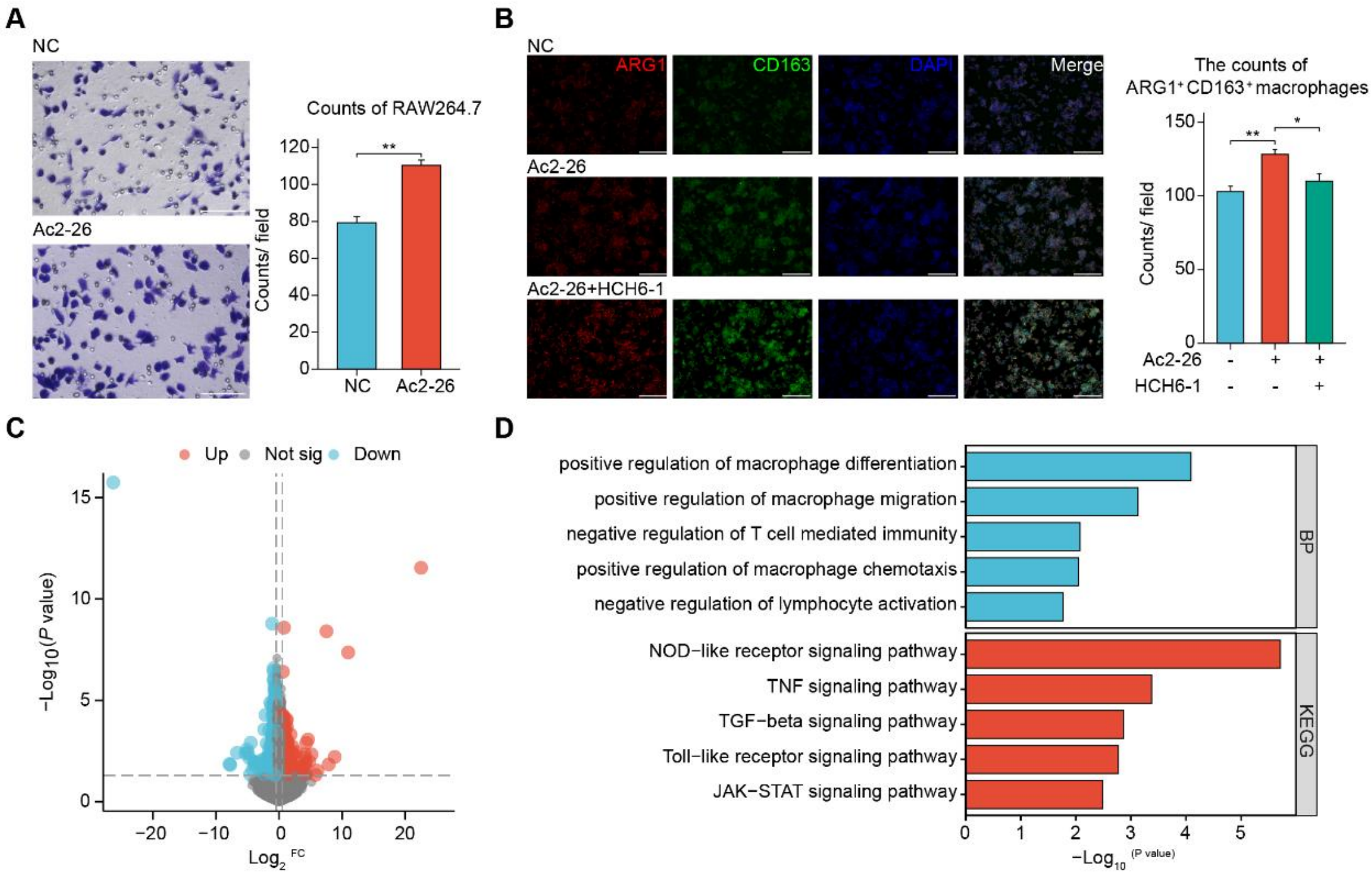

Figure S16

The recruitment and polarization of macrophages regulated by $CD4^{+}$ $ANXA1^{+}$ T_{cm}

(A) Migration ability of macrophages and Ac2-26-stimulated macrophages assessed by the transwell assay (Scale: 100μm).

(B) Phenotypic analysis of macrophages via IF staining (Scale: 100μm; In vitro experiment, n = 15).

(C) The different expressed genes of RAW264.7 and Ac2-26 treated RAW264.7 based on bulk RNA-seq.

(D) The GO and KEGG analysis of macrophage-related enriched signaling pathways based on bulk RNA-seq.

NC, negative control; KEGG, Kyoto Encyclopedia of Genes and Genomes; GO, Gene Ontology; BP, Biological Process

* $p<0.05$, ** $p<0.01$, *** $p<0.001$, **** $p<0.0001$

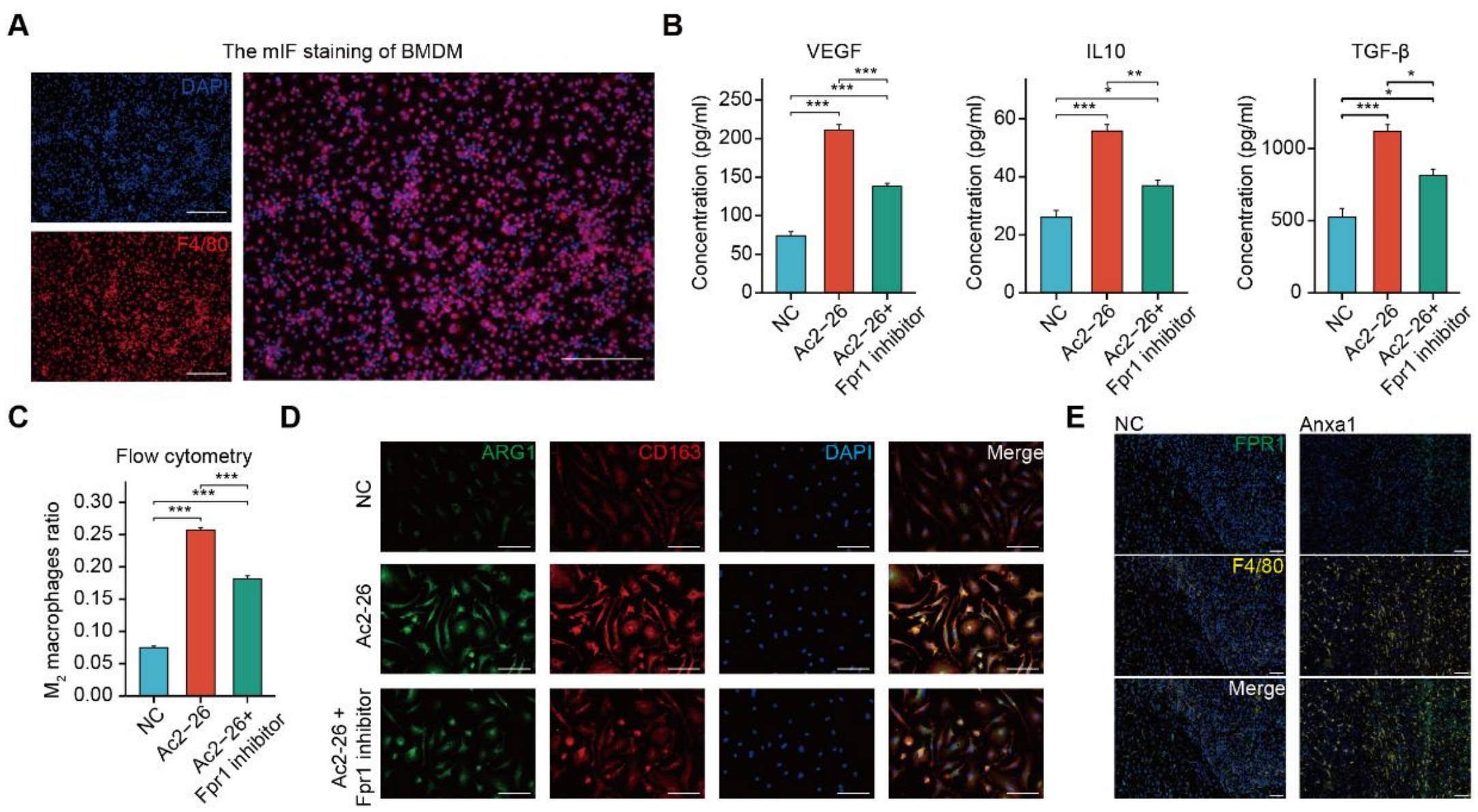

Figure S17

The polarization of BMDM regulated by ANXA1-FPR1 signaling pathway

(A) The identification of BMDM via mIF staining (Scale: 100μm; In vitro experiment).

(B) Cytokine expression of VEGF, IL-10 and TGF-β determined by ELISA.

(C) Flow cytometry of M_2-type macrophages in different groups, respectively.

(D) The mIF staining of M_2-type macrophages in different groups, respectively (Scale: 50μm; In vitro experiment).

(E) The mIF staining of $FPR1^+$ macropahegs in margin zones of iCCA in NC group and Ac2-26 treated group, respectively (Scale: 50μm; In vivo experiment, n = 10).

NC, negative control

* p<0.05, ** p<0.01, *** p<0.001, **** p<0.0001

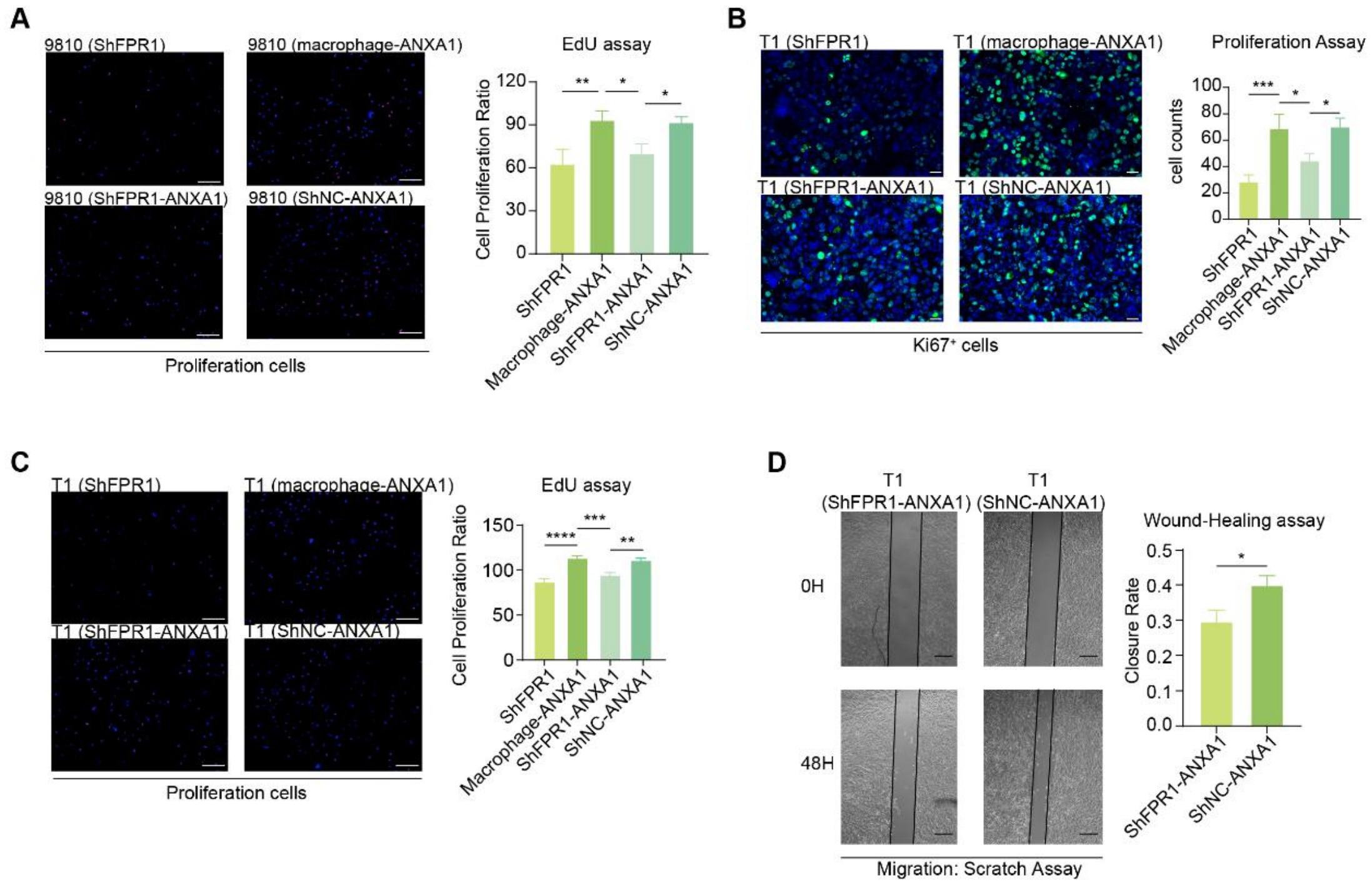

Figure S18

(A) Proliferation ability of HCCC-9810 in control groups and treated groups via EdU assay (Scale: 100μm; In vitro experiment).

(B) Quantitative analysis of Ki67⁺ cells (Scale: 20μm; In vitro experiment).

(C) Proliferation ability of HuCCT1 in control groups and treated groups via EdU assay (Scale: 100μm; In vitro experiment).

(D) Migration ability of HuCCT1 in control groups and treated groups via scratch-wound healing assay. (Scratch-wound healing assay, Scale: 100μm)

* $p<0.05$, ** $p<0.01$, *** $p<0.001$, **** $p<0.0001$

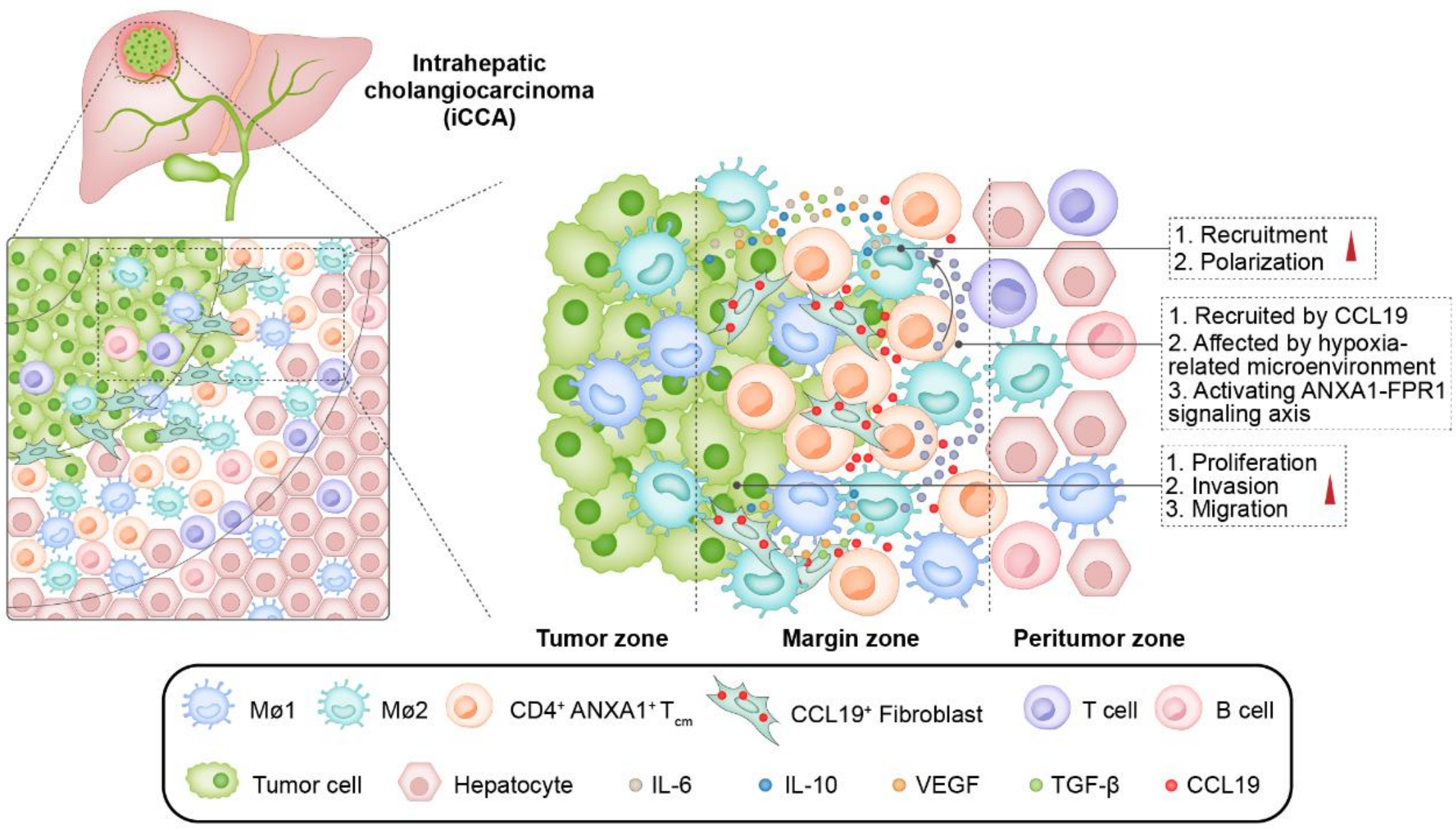

Figure S19

Schematic diagram of immune microenvironment in the tumor margin.

Schematic diagram showing that enriched $CD4^+$ $ANXA1^+$ T_{cm} in the tumor margin recruited macrophages and promoted macrophages polarization into M2 phenomenon, accelerating the tumor progression.

M1, M_1-type macrophage; M2, M_2-type macrophage; T_{cm}, central memory T cell

Table S2

Variable	Total iCCA (n=45)
**Age, mean (SD)**	57.4 (11.2)
**Sex, male/female, n (%)**	26/19 (57.8%/42.2%)
**Primary diseases**	
**iCCA**	45
**Preoperative basic conditions**	
**HBsAg positivity, n (%)**	9 (20.0%)
**CEA, mean (SD)**	10.7(22.4)
**CA199, mean (SD)**	799.3(1829.4)
**AFP, mean (SD), ng/mL**	13.2 (0.8)
**Albumin, mean (SD), g/L**	41. 6(4.2)
**TB, mean (SD)**	16.1 (20.6)
**ALT, mean (SD)**	37.3(49.1)
**PT, mean (SD)**	11.4 (0.8)
**AST, mean (SD)**	33.1 (34.1)
**Liver cirrhosis, n (%)**	
**No cirrhosis**	26(57.8%)
**Mild**	7(15.6%)
**Moderate**	7(15.6%)
**Severe**	5(11%)
**Tumor size, mean (SD)**	7.3(3.7)
**Tumor number, n (%)**	
**Single**	35 (77.8)
**>1**	10 (22.2)
**MVI, n (%)**	17(37.8%)
**Lymph node metastasis, n (%)**	22(48.9%)
**Preoperative tumor metastasis, n (%)**	10(22.2%)

1. Han G, Spitzer M, Bendall S, Fantl W, Nolan G. Metal-isotope-

tagged monoclonal antibodies for high-dimensional mass cytometry. Nature protocols. 2018; 13: 2121 - 48.
2. Lim C, Lee Y, Pan L, Lai L, Chua C, Wasser M, et al. Multidimensional analyses reveal distinct immune microenvironment in hepatitis B virus-related hepatocellular carcinoma. Gut. 2019; 68: 916 - 27.
3. Butler A, Hoffman P, Smibert P, Papalexi E, Satija R. Integrating single-cell transcriptomic data across different conditions, technologies, and species. Nature biotechnology. 2018; 36: 411 - 20.
4. Stuart T, Butler A, Hoffman P, Hafemeister C, Papalexi E, Mauck W, et al. Comprehensive Integration of Single-Cell Data. Cell. 2019; 177: 1888 - 902.e21.
5. Aran D, Looney A, Liu L, Wu E, Fong V, Hsu A, et al. Reference-based analysis of lung single-cell sequencing reveals a transitional profibrotic macrophage. Nature immunology. 2019; 20: 163 - 72.
6. Aibar S, González-Blas C, Moerman T, Huynh-Thu V, Imrichova H, Hulselmans G, et al. SCENIC: single-cell regulatory network inference and clustering. Nature methods. 2017; 14: 1083 - 6.
7. Chen L, Yandong G, Han Z, Hua Y, Yang Z, Zhilei B, et al. Delineating spatiotemporal and hierarchical development of human fetal innate lymphoid cells. Cell Res. 2021; 31.
8. Trapnell C, Cacchiarelli D, Grimsby J, Pokharel P, Li S, Morse M, et al. The dynamics and regulators of cell fate decisions are revealed by pseudotemporal ordering of single cells. Nature biotechnology. 2014; 32: 381 - 6.
9. Vento-Tormo R, Efremova M, Botting R, Turco M, Vento-Tormo M, Meyer K, et al. Single-cell reconstruction of the early maternal-fetal interface in humans. Nature. 2018; 563: 347 - 53.
10. Xiong A, Zhang J, Chen Y, Zhang Y, Yang F. Integrated single-cell transcriptomic analyses reveal that GPNMB-high macrophages promote PN-MES transition and impede T cell activation in GBM. EBioMedicine. 2022; 83: 104239.
11. Vipul S, Nigel C, Joseph L, Yifei Y, Jinyue L, Wan Kee C, et al. BANKSY unifies cell typing and tissue domain segmentation for scalable spatial omics data analysis. Nat Genet. 2024; 56.
12. Linyu G, Xiaojun T, Kangxing Z, Dandan W, Shiying W, Genhong Y, et al. MicroRNA-663 induces immune dysregulation by inhibiting TGF-β1 production in bone marrow-derived mesenchymal stem cells in patients with systemic lupus erythematosus. Cell Mol Immunol. 2019; 16.
13. Yunhong T, Chunshan L, Zhiyong L, Meiling A, Baiyao W, Kunpeng D, et al. Exosomal B7-H4 from irradiated glioblastoma cells contributes to increase FoxP3 expression of differentiating Th1 cells and promotes tumor growth. Redox Biol. 2022; 56.

14. Garelli A, Heredia F, Casimiro A, Macedo A, Nunes C, Garcez M, et al. Dilp8 requires the neuronal relaxin receptor Lgr3 to couple growth to developmental timing. Nature communications. 2015; 6: 8732.
15. Zhang K, Yao E, Chuang E, Chen B, Chuang E, Chuang P. mTORC1 signaling facilitates differential stem cell differentiation to shape the developing murine lung and is associated with mitochondrial capacity. Nature communications. 2022; 13: 7252.
16. Xia Z, Ricardo G, David M M. The isolation and characterization of murine macrophages. Curr Protoc Immunol. 2008.
17. Yang C, Yuan W, Yang X, Li P, Wang J, Han J, et al. Circular RNA circ-ITCH inhibits bladder cancer progression by sponging miR-17/miR-224 and regulating p21, PTEN expression. Molecular cancer. 2018; 17: 19.
18. Zhang Y, Liu G, Zeng Q, Wu W, Lei K, Zhang C, et al. CCL19-producing fibroblasts promote tertiary lymphoid structure formation enhancing anti-tumor IgG response in colorectal cancer liver metastasis. Cancer cell. 2024; 42: 1370-85.e9.
19. Pei G, Yao Y, Yang Q, Wang M, Wang Y, Wu J, et al. Lymphangiogenesis in kidney and lymph node mediates renal inflammation and fibrosis. Science advances. 2019; 5: eaaw5075.
20. Yu W, Sheng-Nan S, Qing L, Yang-Yang Y, Jian G, Kun W, et al. Autocrine Complement Inhibits IL10-Dependent T-cell-Mediated Antitumor Immunity to Promote Tumor Progression. Cancer Discov. 2016; 6.
